# Insulin-like growth factor I mitigates post-traumatic stress by inhibiting AMP-kinase in orexin neurons

**DOI:** 10.1038/s41380-022-01442-9

**Published:** 2022-02-03

**Authors:** M. Estrella Fernández de Sevilla, Jaime Pignatelli, Jonathan A. Zegarra-Valdivia, Pablo Mendez, Angel Nuñez, Ignacio Torres Alemán

**Affiliations:** 1 Cajal Institute (CSIC), Madrid, Spain; 2 Ciberned, Madrid, Spain; 3 Neuroscience Postgraduate Program, UAM, Madrid, Spain; 4 Achucarro Basque Neuroscience Center, Leioa, Spain; 5 Department of Anatomy, Histology and Neuroscience, School of Medicine, UAM, Madrid, Spain; 6 IKERBASQUE, Basque Foundation for Science, Bilbao, Spain

**Keywords:** Neuroscience, Physiology

## Abstract

Maladaptive coping behaviors are probably involved in post-traumatic stress disorders (PTSD), but underlying mechanisms are incompletely understood. We now report that mice lacking functional insulin-like growth factor I (IGF-I) receptors in orexin neurons of the lateral hypothalamus (Firoc mice) are unresponsive to the anxiolytic actions of IGF-I and develop PTSD-like behavior that is ameliorated by inhibition of orexin neurons. Conversely, systemic IGF-I treatment ameliorated PTSD-like behavior in a wild-type mouse model of PTSD (PTSD mice). Further, systemic IGF-I modified the GABA/Glutamate synaptic structure in orexin neurons of naïve wild-type mice by increasing the dephosphorylation of GABA(B) receptor subunit through inhibition of AMP-kinase (AMPK). Significantly, pharmacological inhibition of AMPK mimicked IGF-I, normalizing fear behavior in PTSD mice. Thus, we suggest that IGF-I enables coping behaviors by balancing E/I input onto orexin neurons in a context-dependent manner. These observations provide a novel therapeutic approach to PTSD through modulation of AMPK.

## Introduction

Resilience and vulnerability to mood disturbances rely on coping strategies that purportedly show consistent behavioral and neuroendocrine patterns [1]. In this regard, it is known that exposure to a traumatic event, that may affect a large part of the world population, will trigger post-traumatic stress disorder (PTSD) only in a relatively small proportion of exposed individuals, probably because of maladaptive coping [2]. Worryingly, specific subsets of the population are at greater risk, such as those exposed to conflicts, because of greater exposure to trauma [3]. Unfortunately, treatment of PTSD remains largely unsatisfactory [4]. Therefore, knowledge of the mechanisms of vulnerability and resilience to stress disorders is crucial to develop treatments, and even to prevent these illnesses.

We recently found that the neuroactive hormone IGF-I is associated to vulnerability to stress [5] and are now exploring underlying brain circuits to determine whether IGF-I is a neuroendocrine modulator of coping behaviors. Indeed, regulatory actions of IGF-I on mood are increasingly recognized, pointing to a potential role in coping strategies [5–9]. The relation of circulating IGF-I with mood encompasses multiple angles, such as anxiolysis [5, 9, 10], arousal [11], playfulness [12], or depression [6, 13]. The latter aspect is profusely documented in the literature, attesting to the increasing recognition of the relevance of IGF-I in mood disorders.

In search for possible cellular targets of IGF-I in mood modulation, we focused on the hypothalamus, a brain area that expresses IGF-I receptors (http://mouse.brain-map.org/experiment/show?id=69735263), is involved in fear memories [14], and is central in neuroendocrine regulation of stress [15]. Within the hypothalamus, orexin neurons, that express IGF-I receptors [16] and insulin-binding protein 3 (that controls IGF bioavailability) [17], mediate regulatory actions of IGF-I on sleep architecture [16]. In addition, orexin neurons are involved in responses to stress [18], and fear [19], and pharmacological modulation of their activity has been proposed as possible therapy for PTSD [20–22]. After exposure to trauma, PTSD patients show altered coping with stress, developing abnormally long memories of the traumatic event, generalized fear, and other psychological (hyperarousal, anhedonia) and physiological (exaggerated autonomic and stress hormone responses) hyperreactivities [23]. Orexin neurons are also involved in mood regulation in general [24–26], and mood-related traits such as motivated behaviors [27, 28], or arousal [29]. Recently, orexin neurons have been shown to be directly involved in PTSD-like responses in rats [22]. Thus, we hypothesized that orexin circuits may participate in stress-related behaviors modulated by IGF-I.

In this work, we aimed to determine the role of IGF-I as an anxiolytic signal and the role played by orexin neurons. We used mice lacking IGF-I receptors in orexin neurons (Firoc mice) that develop PTSD-like behavior after classical fear conditioning. This is a behavioral paradigm widely used to mimic PTSD [23], as it captures several of its psychological hyperreactivities mentioned above, including disturbed fear learning.

## Results

### Anxiolytic actions of IGF-I involve orexin neurons

**Fig. 1:**
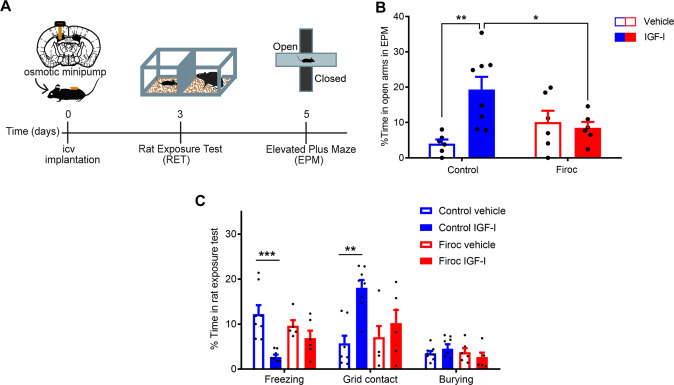
Anxiolytic actions of IGF involve orexin neurons. **A** Time-course of experiments. Day 0: placement of intracerebroventricular minipumps. Day 3: rat exposure test (RET), and ethogram. Day 5: elevated plus maze (EPM). **B** Anxiety levels were measured 2 days after RET in the EPM test as percentage of time spent in the open arms. Only IGF-I-treated control animals spent more time in the open arms (*n* = 6–8 mice/group; **p < 0.01; Two-way-ANOVA followed by Tukey’s multiple comparisons test), while IGF-I-treated Firoc mice did not (*n* = 6–8; *p < 0.05 vs IGF-I treated control littermates). **C** Ethogram obtained during RET. Intracerebroventricular administration of IGF-I attenuated fear behaviors in control animals but not in Firoc mice; freezing was significantly decreased, and grid contacts increased. Burying behavior was not altered by predator exposure (*n* = 6–8 mice/group; **p < 0.01 for grid contact and ***p < 0.001 for freezing; Two-way-ANOVA followed by Tukey’s multiple comparisons test). Mean ± SEM *p < 0.05; **p < 0.01; and ***p < 0.001; in this and following figures.

IGF-I modulates the activity of orexin neurons [16] and reduces anxiety in mice exposed to a predator [5], a natural anxiogenic stimulus [30]. Since orexin neurons are involved in anxiety responses to predator fear [19], we determined whether they participate in anxiolysis by IGF-I. Mice with knocked down IGF-IR in orexin neurons (Firoc mice) were implanted with icv osmotic mini pumps with IGF-I (1 µg/day, 7 days) before exposure to a rat, and predator-elicited anxiety was subsequently evaluated using various behavioral measures (Fig. 1A). We investigated anxiety by time spent in the open arms of the elevated plus maze (EPM), as increased time in an open space reflects lower anxiety levels [31]. While both Firoc and littermates responded similarly to rat exposure (*n *= 6–8 group; Two-way-ANOVA, F (genotype) = 0.7030, *P *= 0.4108), after IGF-I treatment, anxiety was reduced in littermates compared to vehicle-treated ones (Two-way ANOVA, F (treatment) = 5.698, *P *= 0.0260; F (interaction) = 8.740, *P *= 0.0073; followed by Tukey’s multiple comparisons test, q (control vehicle vs control IGF-I) = 2.051, *p* = 0.0039), but not in Firoc mice. Indeed, Firoc mice treated with IGF-I show increased freezing after rat exposure compared to control mice treated with IGF-I (q (control IGF-I vs Firoc IGF-I) = 3.928, *p* = 0.0499; Fig. 1B). Other parameters measuring fear/anxiety behavior during predator exposure such as freezing (*n* = 6–8 mice/group; Two-way-ANOVA, F (genotype) = 0.2673, *P *= 0.6101; F (treatment) = 16.21, *P *= 0.0005; F (interaction) = 5.006, *P *= 0.0352; followed by Tukey’s multiple comparisons test; q (control vehicle vs control IGF-I) = 6.956, p = 0.0003; Fig. 1C) and grid contacts  (*n* = 6–8 mice/group; Two-way-ANOVA, F (genotype) = 2.250, *P* = 0.1467; F (treatment) = 12.86, *P *= 0.0015; F (interaction) = 4.555, *P *= 0.0432; followed by Tukey’s multiple comparisons test; q (control vehicle vs control IGF-I) = 6.178, *p *= 0.0011; 0.0015 Fig. 1C) were also significantly ameliorated by IGF-I in control, but not in Firoc mice. Näive Firoc mice show normal anxiety levels (*n* = 24–30 mice/group; Mann Whitney U Test; U = 337, *p* = 0.6944; Suppl Fig. 1A), and normal latency to escape to an aversive electric shock (*n* = 5–8 mice/group; t-test, *t* = 0.9675, *p* = 0.3541; Suppl Fig. 1B). Collectively, these observations suggest that orexin neurons are involved in modulation of coping behavior (i.e.,: response to predator exposure) by IGF-I.

### IGF-I modulates orexin responses to fear

**Fig. 2:**
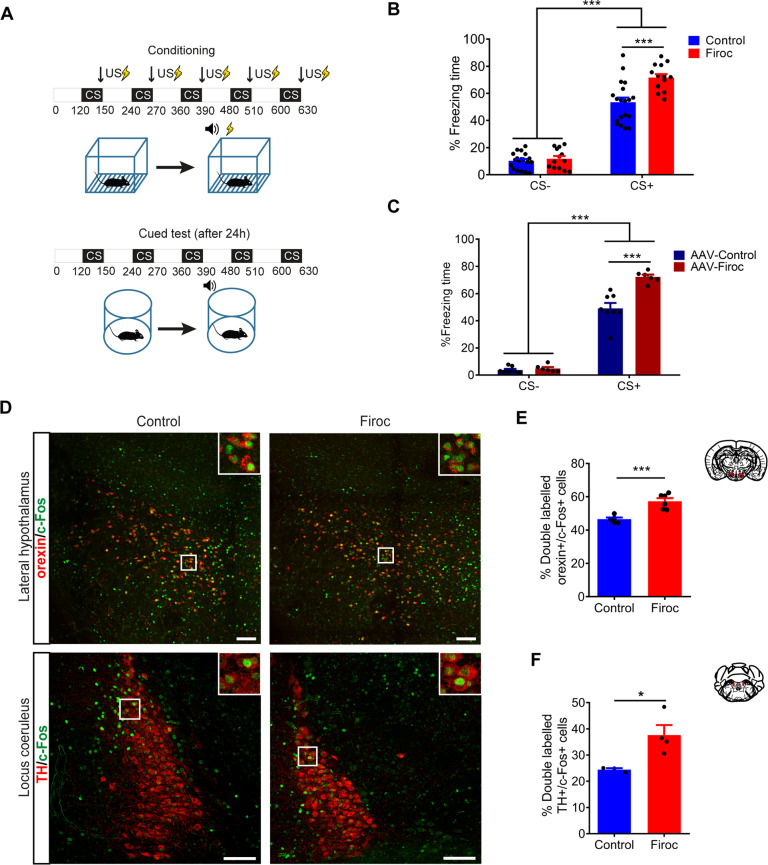
IGF-I modulates fear learning through orexin neurons. **A** Schematic drawing of the cued fear-conditioning protocol. Top: A neutral conditioned stimulus (CS), a tone of 80 dB, is presented together with an aversive unconditioned stimulus (US), an electrical foot-shock of 0.3 mA. Bottom: after US–CS pairing, a new exposure to the CS in the absence of the US in a different context elicits a conditioned freezing response. **B** Firoc mice presented a higher percentage of freezing time in the cued test phase during CS periods (see Supplementary Figure 1C, D for additional information; *n* = 14-19 mice/group; ***p < 0.001; Two-way-ANOVA followed by Tukey’s multiple comparisons test). **C** AAV-Firoc mice display significantly longer freezing time in the cued phase as compared to AAV-Control mice (*n* = 6-8 mice/group; ***p < 0.001; Two-way-ANOVA followed by Tukey’s multiple comparisons test). **D** Upper micrographs: double-stained c-fos (green with Alexa Fluor 488)/orexin cells (red with Alexa Fluor 594) in the lateral hypothalamus of control (left) and Firoc mice (right) exposed to fear conditioning. Lower micrographs: double-stained c-fos (green with Alexa Fluor 488)/tyrosine hydroxylase (TH) cells (red with Alexa Fluor 594) in the locus coeruleus (LC) of control (left) and Firoc mice (right) exposed to fear conditioning. **E** Number of c-fos^+^ /orexin^+^ cells (expressed as percent of total orexin^+^ cells) was significantly increased in Firoc mice after fear conditioning (*n* = 5–6 mice/group; ***p < 0.001; t-test). **F** Number of c-fos^+^ /TH^+^ cells (expressed as percent of total TH^+^ cells) was significantly increased in Firoc mice after fear conditioning (*n* = 3–4 mice/group; *p < 0.05; Welch’s t-test). Scale bars: 100μm.

Based on the above observations, we hypothesized that Firoc mice could show aberrant fear learning. Hence, we submitted them to fear conditioning (Fig. 2A), and found that Firoc mice showed increased fear responses, as measured by time spent in freezing behavior (*n* = 14–19 mice/group; Two-way-ANOVA, F (genotype) = 13.99, *P *= 0.0004; F(stimulus) = 380.6, *P *< 0.0001; F (interaction) = 10.04, *P* = 0.0024, followed by Tukey’s multiple comparisons test, q (control CS+ vs Firoc CS+) = 6.985, *p* < 0.0001; Fig. 2B; and Suppl Fig. 1C, D). To confirm that knock down of IGF-IR in orexin neurons underlies aberrant fear learning, we knocked it down in orexin neurons through viral transduction. pAAV-Orexin-Cre-EGFP virus (AAV-Firoc) or a AAV-CMV-EGFP virus (AAV CMV-Control) were bilaterally injected to adult IGF-IR^f/f^ mice or control littermates (AAV Or-Cre- Control, Suppl Fig. 2A) to produce an inactive (truncated) IGF-IR, confirmed by measuring the expression of intact IGF-IR in AAV-Firoc mice (Suppl Fig. 2B). We then determined freezing behavior after the same protocol of fear learning followed in transgenic Firoc mice. An increased freezing response was observed in AAV-Firoc mice, as compared to pooled control mice (AAV-CMV Control + AAV Or-Cre-Controls; *n* = 6–8 mice/group, Two-way-ANOVA, F (genotype) = 23.93, *P* < 0.0001; F (stimulus) = 521.2, *P *< 0.0001; F (interaction) = 19.77, *P* = 0.0002; followed by Tukey’s multiple comparisons test, q (AAV-Control CS+ vs AAV-Firoc CS+) = 9.338, *p* < 0.0001; Fig. 2C), corroborating findings in Firoc mice.

**Fig. 3:**
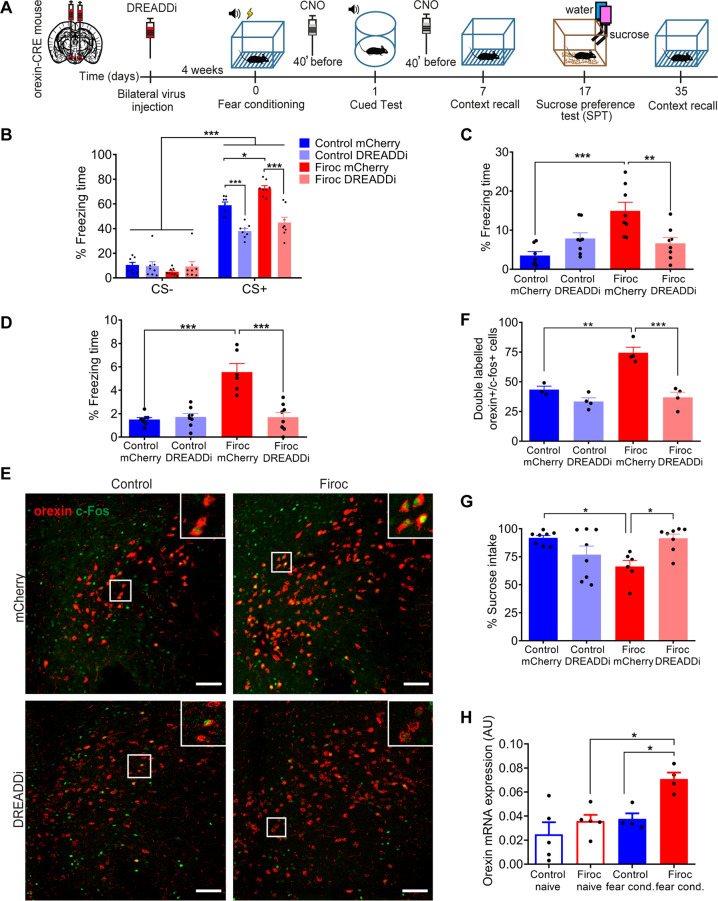
Firoc mice show abnormal fear learning and PTSD-like traits. **A** Time-course of experimental procedures followed in DREADD experiments. **B** Chemogenetic inhibition of orexin neurons in Firoc mice attenuates abnormal freezing responses in cued test day. Control littermates and Firoc mice injected with mCherry control virus show normal and increased fear learning, respectively. All animals received CNO injections 40 min before behavioral testing (*n* = 7–8 mice/group; ***p < 0.001 and *p < 0.05; Three-way-ANOVA followed by Tukey’s multiple comparisons test). **C** One week after fear conditioning, animals were retested to determine extinction recall by placing them in the same context. Firoc mice injected with mCherry show reduced extinction that was normalized after DREADD inhibition (*n* = 7–8 mice/group; ***p < 0.001 and **p < 0.01; Two-way-ANOVA followed by Tukey’s multiple comparisons test). **D** Five weeks after fear conditioning, Firoc mice still show increased freezing responses to the conditioned stimulus (*n* = 6–8 mice/group; ***p < 0.001; Two-way-ANOVA followed by Tukey’s multiple comparisons test). Note that freezing times decay in C and D as compared to Fig. 3B, indicating extinction along time of the learned response in all cases. **E** While c-fos^+^/orexin^+^ cells in Firoc mice are still significantly elevated 5 weeks after fear learning, DREADD inhibition led to normalization of c-fos expression in orexin neurons. Representative immunostainings of double-stained c-fos (green with Alexa Fluor 488)/orexin cells (pseudo-color in red with Alexa Fluor 647) are shown. Insets show magnifications from white squares. Scale bar: 100 μm. **F** Quantification histograms of double labeled c-fos^+^/orexin^+^ cells (*n* = 3–4 mice/group; ***p < 0.001 and **p < 0^.^01; Two^-^way-ANOVA followed by Tukey’s multiple comparisons test). **G** Firoc mice developed anhedonia after fear conditioning, as tested in the sucrose preference test (SPT). Anhedonia was ameliorated by chemogenetic inhibition of orexin neurons (*n* = 6–8 mice/group; *p < 0.05; Two-way-ANOVA followed by Tukey’s multiple comparisons test). **H** Firoc mice show increased orexin mRNA expression after fear learning (*n* = 4–7 mice/group; *p < 0.05; Two-way-ANOVA followed by Sidak’s multiple comparisons test).

After fear learning, Firoc mice also showed increased number of activated orexin (double-labeled c-fos^+^/orexin^+^ neurons; *n* = 5–6 mice/group, *t* = 4.884, *p* = 0.0009, t-test; Fig. 2D, upper panels and Fig. 2E), and locus coeruleus (LC) neurons (double-labeled c-fos^+^/tyrosine hydroxylase^+^ cells; *n* = 3–4 mice/group; Welch’s t-test; *t* = 3.446, *p* = 0.0388; Fig. 2D, lower panels and Fig. 2F), a key downstream connection of orexin neurons in fear responses [32]. Since these observations suggested increased activation of orexin neurons in Firoc mice after fear conditioning, we inhibited orexin activity using chemogenetic inhibition with Designer Receptors Exclusively Activated by Designer Drugs (DREADD). After bilateral injection of an inhibitory AAV-hSyn-DIO-hM4D(Gi)-mCherry virus (hM4Di) into the lateral hypothalamus of Orexin-Cre (controls) and Firoc mice, that resulted in a similar percentage of infected orexin neurons in both groups (Suppl Fig. 2C), we administered CNO to fear-conditioned mice 40 min before behavioral testing (Fig. 3A). After confirming CNO efficacy to inhibit the activity of DREADDi+ orexin neurons (see methods, Suppl Fig. 3), we found that CNO-injected DREADD-Firoc mice normalized their fear responses (*n* = 7–8 mice/group; Three-way-ANOVA, F (virus) = 28.10, *P* < 0.0001; F (genotype) = 3.441, *P *= 0.0742; F (stimulus) = 443.4, *P* < 0.0001, F (stimulus × virus) = 36.76, *P* < 0.0001; F (stimulus × genotype) = 11.01; *P *= 0.0025; F (virus × genotype) = 0.04735, *P* = 0.8293; F (stimulus × virus × genotype) = 2.266, *P* = 0.1435; followed by Tukey’s multiple comparisons test, q (Firoc mCherry CS+ vs Firoc DREADDi CS+) = 9.506, *p* < 0.0001; Fig. 3B). Control littermates and Firoc mice injected with a control mCherry virus show normal and exaggerated fear responses, respectively, as expected (q (Control 14 mCherry CS+ vs Firoc mCherry CS+) = 4.876, *p* = 0.0333; Fig. 3B). These data suggest that orexin neurons in Firoc mice submitted to fear conditioning are over-activated.

### Over-activation of orexin neurons is involved in PTSD-like behavior

Aberrant response to classical fear learning is currently considered a good model of PTSD-like behavior [2], as it provides construct, face and predictive validity [23]. Taking advantage that chemogenetic inactivation of orexin neurons in Firoc mice rescued exaggerated fear responses, we examined the possible development of other PTSD-like traits and its attenuation with DREADD inhibition. Extinction of context-dependent fear responses (context recall) 1 week after fear learning, was markedly impaired in Firoc mice injected with mCherry virus, as compared to mCherry-injected littermate controls (*n* = 7–8 mice/group; Two-way-ANOVA, F (virus) = 1.459, *P* = 0.2376; F (genotype) = 9.773, *P *= 0.0042; F (interaction) = 15.00, *P *= 0.0006; followed by Tukey’s multiple comparisons test, q (Control mCherry vs Firoc mCherry) = 6.882, *p* = 0.0002; Fig. 3C). This indicates abnormal retention of learned fear, a trait seen in PTSD patients [33]. However, after CNO administration, Firoc mice injected with hM4Di virus show normalized fear responses (q (Firoc mCherry vs Firoc DREADDi) = 5.171, *p* = 0.0057; Fig. 3C). Since long-term fear memory is abnormally maintained in PTSD, we re-assessed fear responses to the context in Firoc mice 5 weeks later and found them to be still elevated (*n* = 6–8 mice/group; Two-way-ANOVA, F (virus) = 20.58, *P* = 0.0001, F (genotype) = 25.12, *P* < 0.0001; F (interaction) = 25.87, *P *< 0.0001; followed by Tukey’s multiple comparisons test, q (Control mCherry vs Firoc mCherry) = 9.731, *p* < 0.0001; Fig. 3D). However, Firoc DREADD mice treated with CNO 1 week after fear learning (see Fig. 3A) show normal freezing behavior in the fifth week (q (Firoc 4 mCherry vs Firoc DREADDi) = 9.272, *p* < 0.0001; Fig. 3D). Accordingly, c-fos expression in orexin neurons was still significantly greater 5 weeks after fear conditioning in control (mCherry) Firoc mice, but normal in Firoc DREADD mice treated with CNO at week 1 (*n* = 3–4 mice/group; Two-way-ANOVA, F (virus) = 35.32, *P* < 0.0001; F (genotype) = 18.61, *P  *= 0.0012; F (interaction) = 11.83, *P *= 0.0055; followed by Tukey’s multiple comparisons test, q (Control mCherry vs Firoc mCherry) = 7.472, *p* = 0.0013 and q (Firoc mCherry vs Firoc DREADDi) = 9.766, *p* = 0.0001; Fig. 3E, F).

Anhedonia is another core PTSD trait that can be reliably modelled in mice. Firoc mice injected with mCherry control virus also developed anhedonia after fear conditioning, as measured by the sucrose-preference test (*n* = 6–8 mice/group; Two-way-ANOVA, F (virus) = 0.9174, *P* = 0.3470; F (genotype) = 1.084, *P* = 0.3073; F (interaction) = 14.55, *P* = 0.0008; followed by Tukey’s multiple comparisons tes; q (Control mCherry vs Firoc mCherry) = 4.679, *p* = 0.0137; Fig. 3G). However, anhedonia was ameliorated in Firoc mice chemogenetically inhibited 1 week after fear-learning (q (Firoc mCherry vs Firoc DREADDi) = 4.598, p = 0.0157; Fig. 3G). Naive Firoc mice did not show anhedonia (*n* = 9 mice/group; t-test, *t* = 0.7939, *p* = 0.4389: Suppl Fig. 4A). Interestingly, Firoc mice also showed higher levels than control littermates of orexin in the hypothalamus after fear conditioning (*n* = 4–7 mice/group; Two-way-ANOVA, F (non-conditioned vs conditioned) = 11.14, *P* = 0.0049, F (genotype) = 9.568, *P* = 0.0079; F (interaction) = 2.415, *P* = 0.1425; followed by Sidak’s multiple comparisons test, t (Control conditioned vs Firoc conditioned) = 3.118, *p* = 0.0445; Fig. 3H). These observations suggest that inhibition of over-active orexin neurons early after fear learning is sufficient to block subsequent long-term PTSD-like traits.

### An IGF-I/orexin link in PTSD-like behavior

**Fig. 4:**
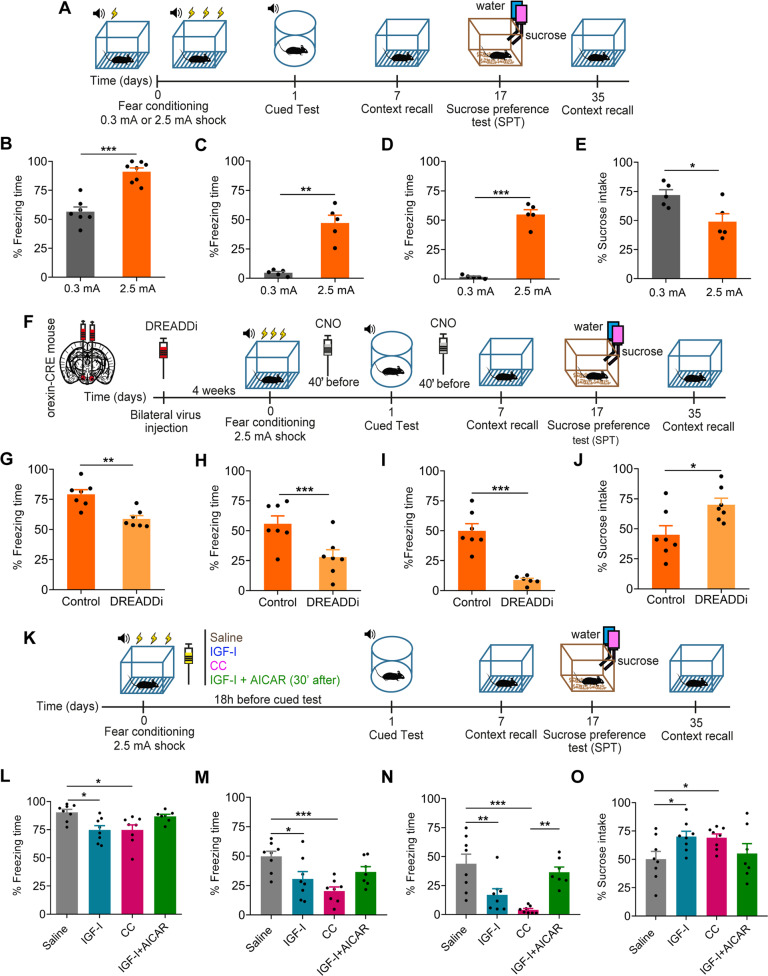
Orexin neurons participate in development of PTSD-like behavior in wild type mice and IGF-I prevent it. **A** Time-course of experimental procedures followed in the PTSD-eliciting, fear learning protocol using 2.5 mA shocks. **B** Wild type mice submitted to this protocol (PTSD mice) develop increased freezing upon re-exposure to the CS (*n* = 7–8 mice/group; ***p < 0.001; t-test). **C** Exaggerated freezing is maintained 1 week later (*n* = 5 mice/group; **p < 0.01; Welch’s *t*-test). **D**, Significantly, longer freezing was seen at 5 weeks after fear learning in PTSD mice (*n* = 5 mice/group; ***p < 0.001; Welch’s *t*-test). **E** Wild type PTSD mice develop anhedonia, as tested in the SPT (*n* = 5 mice/group; *p < 0.01; t-test). **F** Time-course of experimental procedures followed in the PTSD-eliciting protocol using DREADD modulation. **G** Chemogenetic inhibition with DREADDi normalizes freezing responses upon exposure to the CS (*n* = 7 mice/group; **p < 0.001; t-test). **H** Extinction of fear 1 week later was also normalized by DREADDi (*n* = 7 mice/group; **p < 0.01; t-test). **I**, Exaggerated freezing 5 weeks after fear learning was also normalized by DREADD in PTSD mice (*n* = 6–7 mice/group; ***p < 0.001; t-test). **J** Anhedonia developed by PTSD mice was rescued with DREADD-mediated chemogenetic inhibition of orexin neurons (*n* = 7 mice/group; *p < 0.05; t-test). **K**, Time-course of experimental procedures followed in the PTSD-eliciting, fear learning protocol with IGF-I, CC or IGF-1 + AICAR administration. **L** Treatment of wild type PTSD mice with ip IGF-I (1 µg/gr) 6 h after the training session inhibited exaggerated freezing responses in cued test day (*n* = 8 mice/group; *p < 0.05; One-way-ANOVA followed by Tukey’s multiple comparisons test). PTSD mice treated with CC (ip, 10 mg/kg), an inhibitor of AMPK, show amelioration of freezing responses (*n* = 8 mice/group; *p < 0.05; One-way-ANOVA followed by Tukey’s test). PTSD mice treated with AICAR (ip, 500 mg/kg), an activator of AMPK, after IGF-I injection (*n* = 7-8 mice/group; One-way-ANOVA followed by Tukey’s test). Administration of AICAR in these mice abrogated the beneficial effects of IGF-I. **M** Context recall 1 week after training was significantly ameliorated by systemic IGF-I (*n* = 8 mice/group; *p < 0.05; One-way-ANOVA followed by Tukey’s test) and by CC injection (*n* = 8 mice/group; ***p < 0.001; One-way-ANOVA followed by Tukey’s test) but not by IGF + AICAR (*n* = 7–8 mice/group; One-way-ANOVA followed by Tukey’s test). **N** IGF-I ameliorated freezing responses 5 weeks after fear learning in PTSD mice (*n* = 8 mice/group; **p < 0.01; One-way-ANOVA followed by Tukey’s multiple comparisons test). Also, CC diminished freezing responses 5 weeks after fear learning in PTSD mice (*n* = 8 mice/group; ***p < 0.001; One-way-ANOVA followed by Tukey’s multiple comparisons test). IGF + AICAR treated group did not show any amelioration of freezing behavior (*n* = 7–8 mice/group; One-way-ANOVA followed by Tukey’s multiple comparisons test). **O** Anhedonia, as tested in the SPT, was also ameliorated by IGF-I treatment (*n* = 8 mice/group; *p < 0.05; One-way-ANOVA followed by Tukey’s test) and by CC injection (*n* = 8 mice/group; *p < 0.05; t-test) but not by IGF + AICAR (*n* = 7–8 mice/group; One-way-ANOVA followed by Tukey’s test).

**Fig. 5:**
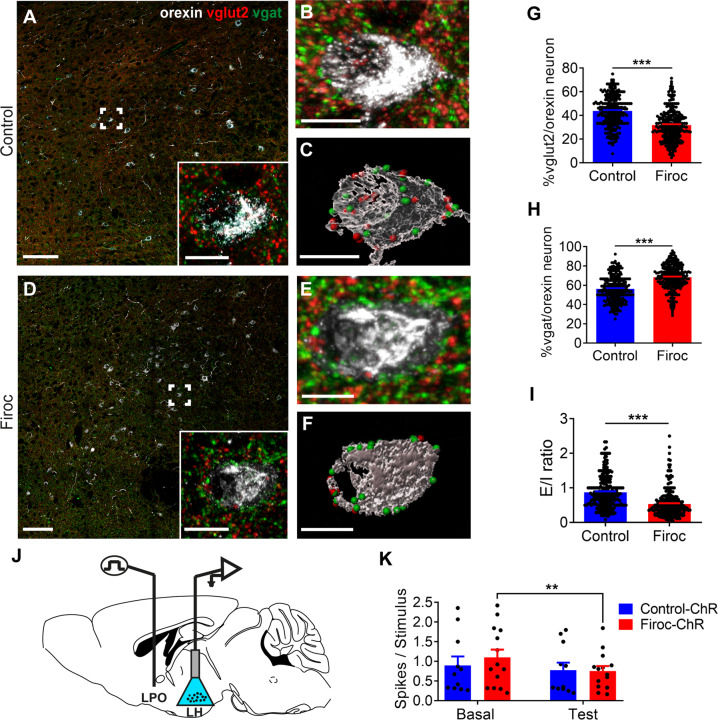
Firoc mice show unbalanced Glutamatergic/GABAergic input onto orexin neurons. **A-F** Representative micrographs of triple immunostaining of Vglut2 (pseudo-color in red with Alexa Fluor 647), Vgat (green with Alexa Fluor 488), and orexin (pseudo-color in white Alexa Fluor 594) in lateral hypothalamus of control (**A-C**) and Firoc (**D-F**) mice revealed a significantly decreased number of Vglut2 puncta together with increased Vgat puncta onto orexin neurons (*n* = 100 orexin neurons/mouse, 3 mice per group; ***p < 0.001; Mann Whitney U Test). **B**, **E** 3D representation of an orexin neuron (from the inset in **A** and **D**) using Imaris software. **C**, **F**, Surface area of the same orexin neurons shown in **B** and **E** showing Vglut (red points) and Vgat spots (green points). **G** Percentage of Vglut2 spots in orexin neurons from each experimental group (*n* = 100 orexin neurons/mouse, 3 mice per group, ***p < 0.001; Mann Whitney U Test). **H** Percentage of Vgat spots (*n* = 100 orexin neurons/mouse, 3 mice per group; ***p < 0.001; Mann Whitney U Test). **I** Excitatory/ inhibitory ratio *(*E*/*I) is significantly decreased in Firoc mice (*n* = 100 orexin neurons/mouse, 3 mice per group); ***p < 0.001; Mann Whitney U Test. Scale bars in **A** and **D**: 100 μm, and in **B**, **C**, **E** and **F**: 15 μm. **J** A cartoon showing the intracranial localization of the electrode of stimulation in LPO (inhibitory inputs) and the optrode (optogenetic blue LED + Tungsten recording electrode) used to identify orexinergic cells in the LH/PeF area. **K** Unitary activity of orexinergic neurons in Or-ChR and Firoc-ChR animals. BLUE light pulses elicited 0.9 ± 0.22 spikes/50 ms in orexinergic neurons of Control-ChR mice, and 1.1 ± 0.19 spikes/50 ms in orexin neurons of Firoc-ChR mice (*n* = 11, and *n* = 14, respectively). Thus, orexin neurons of Control-ChR and Firoc-ChR do not display differences in basal conditions (*p* = 0.7299). However, after LPO stimulation (inhibitory inputs) and optogenetic activation, control-ChR mice show a 13% inhibition (0.78 ± 0.19 spikes/50 ms; *p* = 0.1135), whereas Firoc-ChR display a 31% inhibition (0.76 ± 0.12 spikes/50 ms; **p < 0.01; sex balanced, Two-way RM ANOVA, Sidak’s Multiple comparison tests). Scale bars in **A** and **D**: 100 μm, and in **B**, **C**, **E** and **F**: 15 μm.

Wild type mice display exaggerated freezing after fear learning through exposure to a 2.5 mA electric shock (*n* = 7–8 mice/group; *p* < 0.0001, t-test, *t* = 6.811, *p* < 0.0001; Fig. 4A, B). These mice show a PTSD-like behavior as assessed by prolonged exaggerated freezing one (*n* = 5 mice/group, Welch’s t-test, *t* = 6.327, *p* = 0.0026; Fig. 4C) and five weeks after (*n* = 5 mice/group; Welch’s t-test, *t* = 12.67, *p* = 0.0002; Fig. 4D) and anhedonia (*n* = 5 mice/group; t-test, *t* = 2.780, *p* = 0.0239; Fig. 4E). We then determined the role of orexin neurons in this PTSD-like model [34]. We injected hM4Di or mCherry viruses to orexin-Cre mice submitted to the 2.5 mA shock protocol (Fig. 4F). Increased freezing was ameliorated by DREADD inhibition of orexin neurons after administration of CNO (*n* = 7 mice/group; t-test, *t* = 4.270, *p* = 0.0011; Fig. 4G). Protracted, PTSD-like behavioral responses at 1 (*n* = 7 mice/group; t-test, *t* = 3.104, *p* = 0.0091, Fig. 4H) and 5 weeks after fear learning (*n* = 7 mice/group; t-test, *t* = 6.265, *p* < 0.0001; Fig. 4I), and anhedonia (*n* = 7 mice/group; t-test, *t* = 2.689, *p* = 0.0197; Fig. 4J), were also attenuated by DREADD inhibition in these mice.

Next, we examined with a similar experimental protocol whether IGF-I attenuates PTSD-like responses in wild type mice (Fig. 4K). Indeed, wild type mice that develop exaggerated freezing after fear learning with 2.5 mA shocks, show significantly reduced freezing responses after treatment with systemic IGF-I (ip, 1 µg/gr, 18 h before cued test) as compared to vehicle-treated controls (*n* = 8 mice/group; F (treatment) = 5.720, *P* = 0.0036; One-way-ANOVA followed by Tukey’s multiple comparisons test, q (saline vs IGF-I) = 4.651, *p* = 0.0140; Fig. 4L). Protective effects of systemic IGF-I administration were seen for at least 1 (*n* = 8 mice/group, F (treatment) = 6.826, P = 0.0014; One-way-ANOVA followed by Tukey’s multiple comparisons test,  q (saline vs IGF-I) = 4.066, *p* = 0.0368; Figs. 4M) and 5 weeks (*n* = 8 mice/group; F (treatment) = 11.06, *P* < 0.0001; One-way-ANOVA followed by Tukey’s multiple comparisons test, q (saline vs IGF-I) = 4.950, *p* = 0.0083; Fig. 4N). Anhedonia induced by the PTSD-eliciting protocol in wild type mice was also ameliorated by IGF-I treatment (*n* = 8 mice/group; F (treatment) = 3.836, *P* = 0.0208; One-way-ANOVA followed by Tukey’s multiple comparisons test, q (saline vs IGF-I) = 3.892, *p* = 0.0484; Fig. 4O). Conversely, Firoc mice displaying exaggerated fear responses after fear learning with 0.3 mA electric shocks were unresponsive to IGF-I treatment (*n* = 8 mice/group; F (treatment) = 15.16, *P* < 0.0001; One-way-ANOVA followed by Tukey’s multiple comparisons test, q (Firoc saline vs Firoc IGF-I) = 2.196, *p* = 0.2851; Suppl Fig. 4B). Collectively, these results suggest that either chemogenetic inhibition of orexin neurons or systemic administration of IGF-I after fear learning in PTSD-mice abrogates development of PTSD-like behavior.

### Excitatory/inhibitory balance in orexin neurons is modulated by IGF-I

Orexin neurons show a highly plastic excitatory/inhibitory input that can be hormonally regulated [35]. Thus, we determined whether knock down of IGF-IR activity in orexin neurons of Firoc mice affects Glutamatergic and GABAergic (Glu/GABA) inputs onto them. We combined orexin and Vgat or Vglut2 immunocytochemistry to identify GABAergic or glutamatergic synaptic puncta, respectively. We found that Firoc mice show decreased number of Vglut2 puncta/orexin cell and increased Vgat puncta/orexin cell (*n* = 100 orexin neurons/mouse, 3 mice per group; *p* < 0.0001 for both measures; Mann Whitney U Test, *U* = 33249 and *p* < 0.0001 for Vglut2, *U* = 23,282 and *p* < 0.0001 for Vgat; Fig. 5A–H), resulting in a decreased Glu/GABA ratio onto orexin neurons (*n* = 100 orexin neurons/mouse, 3 mice per group; Mann Whitney U Test, *U* = 17,724, *p* < 0.0001 for both measures; Fig. 5I).

To assess whether a decreased Glu/GABA ratio results in greater inhibition of orexin neurons in Firoc mice, we studied lateral pre-optic area (LPO)-mediated inhibition of orexin neurons [36] in the lateral hypothalamus (LH; Fig. 5J). We used Firoc (Firoc-ChR) and littermate (Control-ChR, control) mice expressing channelrhodopsin in orexin neurons and identified them through optogenetic stimulation (Fig. 5J), as described [16]. Short-lasting blue LED stimuli induced similar increases in spike firing in orexinergic neurons of both experimental groups (Firoc ChR, RM One-way ANOVA, (treatment) = F (1.71, 44.68): 8.199; *p* = 0.0015; Holm-Sidak’s Multiple comparison tests, Basal vs 100: *p* = 0.0034, Basal vs 200: *p *= 0.0162; Control ChR, RM one-way ANOVA, (treatment)= F(1.74, 31.41): 5.149, *p *= 0.0146; Holm-Sidak’s Multiple comparison tests, Basal vs 100: *p* = 0.0032, Basal vs 200: p = 0.044; Suppl Fig. 4C). The effect lasted 200 ms, even though the blue-light pulse lasted 300 ms. Once orexin neurons were optogenetically identified, light pulses onto orexin neurons elicited 0.9 ± 0.22 spikes/50 ms (*n* = 11) in Control-ChR and 1.1 ± 0.19 spikes/50 ms (*n* = 14) in Firoc-ChR. However, electrical stimulation of the LPO 100 ms prior to light stimulation of orexin neurons inhibited their response (Fig. 5K). In orexin neurons of Control-Chr mice, the response to light stimulation was slightly reduced, from 0.9 ± 0.22 spikes/50 ms to 0.78 ± 0.19 spikes/50 ms (13%; *p* = 0.1135; *n* = 11), while in Firoc-ChR orexin neurons the reduction was statistically significant (31%: from 1.1 ± 0.19 spikes/50 ms to 0.76 ± 0.12 spikes/50 ms; *p* = 0.0030; *n* = 14; Two-way RM ANOVA, (time) = F(1, 23): 10.46, *p* = 0.0037; Sidak’s Multiple comparison tests, Control ChR group: *p* = 0.4679, Firoc ChR group: *p* = 0.0030). Collectively, these changes agree with previous observations of decreased responses of orexin neurons to afferent stimulation in Firoc mice [16], and confirm that knocking down IGF-IR activity in orexin neurons enhances inhibitory transmission onto them.

**Fig. 6:**
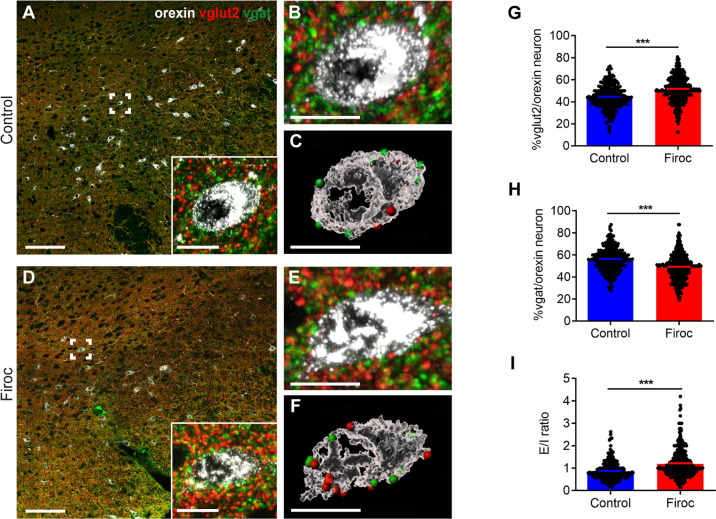
Fear conditioning reverses Glutamatergic/GABAergic input onto orexin neurons in Firoc mice. Representative micrographs of triple immunostaining of Vglut2 (pseudo-color in red with Alexa Fluor 647), Vgat (green with Alexa Fluor 488), and orexin (pseudo color in white with Alexa Fluor 594) in lateral hypothalamus of control (**A-C**) and Firoc (**D-F**) mice after fear learning. **B, E** 3D representations of an orexin neuron (from the inset in panels **A** and **D**) using Imaris software. **C, F** Surface area of the same orexin neurons shown in **B** and **E** showing Vglut (red points) and Vgat spots (green points). **G** Number of Vglut2 puncta onto orexin neurons is increased in Firoc mice after fear learning. **H** Number of Vgat puncta is significantly decreased in Firoc mice (*n* = 100 orexin neurons/mouse, 3 mice per group; ***p < 0.001; Mann–Whitney U Test). **I** Fear learning induces a reversal in the excitatory/inhibitory ratio in Firoc mice (***p < 0.001; Mann–Whitney U Test). Scale bars in **A** and **D**: 100 μm, and in **C**, **D**, **F**, and **G**: 15 μm.

As organization of synaptic architecture of orexin neurons is highly experience-dependent [37], we then assessed whether fear learning in Firoc mice induces synaptic remodeling that could explain the observed upregulation of orexin activity. We observed increased Vglut2 puncta in orexin neurons of Firoc mice undergoing fear learning (*n* = 100 orexin neurons/mouse, 3 mice per group; Mann Whitney U Test, *U* = 55,899, *p* < 0.0001 for both measures; Fig.  6A–G). This agrees with increased hypothalamic Nrg1 expression -that promotes glutamatergic transmission [38], upon fear-learning in Firoc mice (*n* = 4 mice/group; Two-way ANOVA, F (naive vs conditioned) = 45.57, *P* < 0.0001, F (genotype) = 8.382, *P* = 0.0125; F (interaction) = 7.855, *p* = 0.0150; followed by Sidak´s test, t (Firoc naïve vs Firoc conditioned) = 6.940, *p* < 0.0001; Suppl Fig. 4D). At the same time, decreased Vgat puncta in orexin neurons was observed (*n* = 100 orexin neurons/mouse, 3 mice per group; Mann Whitney U Test, *U* = 69820, *p* < 0.0001 for both measures; Fig. 6D–H). Overall, the Glu/GABA ratio was increased in Firoc mice submitted to fear learning (*n* = 100 orexin neurons/mouse, 3 mice per group; Mann Whitney U Test, *U* = 44,813, p < 0.0001 for both measures; Fig. 6I). Increased E/I balance fully agrees with the observed enhanced freezing responses of Firoc mice.

**Fig. 7:**
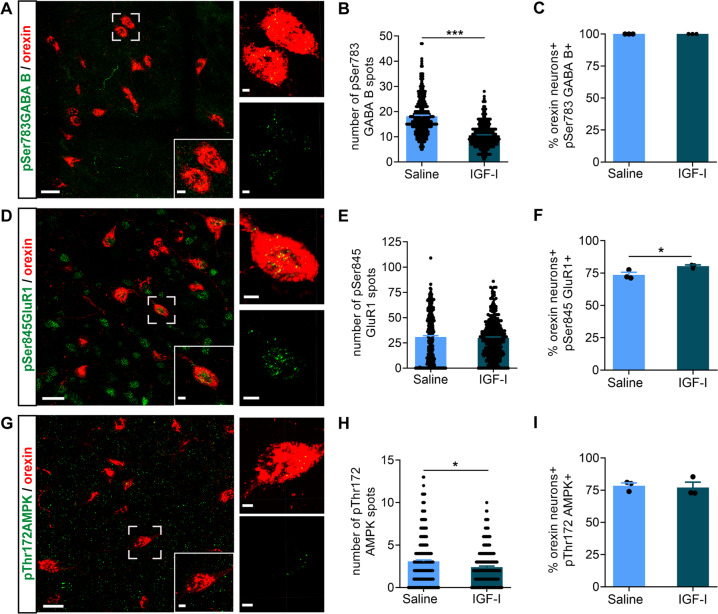
IGF-I modulates GABAergic and Glutamatergic inputs onto orexin neurons. **A-C** Double immunostaining showed that all orexin neurons express pSer^783/893^ GABA(B)R, and that systemic IGF-I treatment (ip 1 µg/gr, 2 h) elicited a significant decrease in the number of pSer^783/893^ GABA(B)R spots (green with Alexa Fluor 488)/orexin cell (red with Alexa Fluor 594; *n* = 100 orexin neurons/mouse, 3 mice per group; ***p < 0.001; Mann–Whitney U Test) Representative micrographs and quantification histograms are shown. **D-F** Double immunostaining of pSer^845^GluR1 receptor and orexin showed a modest but significant increase in the percentage of these neurons expressing the active form of GluR1 after IGF-I treatment (*n* = 100 orexin neurons/mouse, 3 mice per group; *p < 0.05; t-test). No changes in the number of pSer^845^GluR1 receptor spots (green with Alexa Fluor 488)/orexin neuron (red with Alexa Fluor 594) were detected after IGF-I treatment. Representative micrographs and quantification histograms are shown. **G-I** Double immunostaining of pThr^172^ AMPK (green with Alexa Fluor 488) and orexin (red with Alexa Fluor 594) showed reduced number of positive spots/orexin cell after IGF-I treatment without altering the percentage of double-labeled cells (*n* = 100 orexin neurons/mouse, 3 mice per group; *p < 0.05; Mann–Whitney U Test). Representative micrographs and quantification histograms are shown. Scale bars in **A**, **D** and **G**: 25 μm, and 5 μm in magnifications.

Collectively, the above results indicate that the absence of a functional IGF-IR in orexin neurons imbalances glutamatergic and GABAergic inputs onto the soma of these neurons. To confirm an effect of IGF-I on synaptic architecture of orexin neurons, we administered IGF-I (ip, 1 µg/gr, 2 h before sacrifice) to wild type mice and determined immunostaining in orexin cells of pSer^783/893^ GABA(B)R and pSer^845^GluR1, markers of active GABAergic [39] and Glutamatergic [40] receptors, respectively. While all orexin neurons in saline and IGF-I-treated mice presented pSer^783/893^ GABA(B) R immunoreactivity, IGF-I significantly decreased the number of pSer^783/893^ GABA(B) R puncta in them (*n* = 100 orexin neurons/mouse, 3 mice per group; *p* < 0.0001; Mann Whitney U Test; Fig. 7A–C), indicating decreased postsynaptic GABA(B) R activity. At the same time, IGF-I modestly, but significantly increased the number of double-labeled orexin/pSer^845^ GluR1 neurons, indicating increased number of orexin neurons with active postsynaptic glutamate receptors, although the amount of pSer^845^GluR1 staining per cell was not affected (*n* = 100 orexin neurons/mouse, 3 mice per group; *p* = 0.0395; *t* = 3.011, *p* = 0.0395; Fig. 7D-F). Since AMPK stimulates GABAR function [39], and Akt, that is downstream of IGF-I signaling, may inhibit this kinase [41], we determined the levels of pThr^172^ AMPKα immunoreactivity (an indicator of activation of AMPK [42]) in orexin neurons after IGF-I administration and found them decreased (*n* = 100 orexin neurons/mouse, 3 mice per group; Mann–Whitney U Test, *U* = 40,619, *p* = 0.0367; Fig. 7G–I). Since IGF-I did not affect pSer^783/893^ GABA(B) R immunoreactivity in MCH neurons (*n* = 100 MCH neurons/mouse, 3 mice per group; Mann Whitney U Test, *U* = 43,156, p = 0.3851; Suppl Fig. 4E), a neighboring population known to interact with orexin neurons [43], the latter appear to be the target of IGF-I in modulating fear behavior.

To confirm a role of AMPK in the actions of IGF-I onto orexin neurons, we inhibited this kinase by ip administration of Compound C (CC, 10 mg/kg), a drug that crosses the blood-brain barrier. Indeed, CC-treated wild type mice submitted to the PTSD protocol (Fig. 4K) showed normalized fear behavior, thus mimicking the beneficial effects of IGF-I in cued test day (*n* = 8 mice/group; F (3, 27) = 5.720, *P* = 0.0036, One-way-ANOVA followed by Tukey’s multiple comparisons test, *p* = 0.0141; Fig. 4L), 1 (*n* = 8 mice/group, F (3,27) = 6.826, *P* = 0.0014; One-way-ANOVA followed by Tukey’s multiple comparisons test, *p* = 0.0008; Figs. 4M) and 5 weeks (*n* = 8 mice/group; F (3, 27) = 11.06, *P* < 0.0001; One-way-ANOVA followed by Tukey’s multiple comparisons test, *p* = 0.0001; Fig. 4N) after fear conditioning. Anhedonia was also ameliorated with CC treatment (*n* = 8 mice/group; F (3, 27) = 3.836, *P* = 0.0208; One-way-ANOVA followed by Tukey’s multiple comparisons test, *p* = 0.0466; Fig. 4O). Of note, Firoc mice treated with CC also show normal freezing behavior, reinforcing that AMPK inhibition is downstream of IGF-I signaling (*n* = 8 mice/group; F (2, 24) = 15.16, *P* < 0.0001, One-way-ANOVA followed by Tukey’s multiple comparisons test *p* < 0.0001; Suppl Fig. 2D). Conversely, administration of AICAR, an AMPK agonist [44], to IGF-I-treated PTSD mice (Fig. 4K) abolished the protective action of this growth factor (*n* = 8 mice/group, One-way-ANOVA followed by Tukey’s; Fig. 4L-O), corroborating a role of AMPK in IGF-I actions.

## Discussion

Plasticity of synaptic inputs onto orexin neurons has been shown to be under circadian [45], and hormonal regulation [35]. Together with recent results [16], the present observations indicate that peripheral IGF-I, a hormone mostly produced by the liver [46], modulates the activity of orexin neurons by balancing their excitatory/inhibitory transmission in a context-dependent manner. In näive intact mice, IGF-I stimulates the activity of orexin neurons [16] and decreases inhibitory postsynaptic boutons in these neurons, which likely is reflected in increased excitability. Since Firoc mice show a decreased E/I ratio, it seems that IGF-I signaling onto orexin neurons is required to maintain normal excitability levels. Intriguingly, Firoc mice show a markedly increased E/I ratio after fear conditioning compared to controls. Thus, control mice show a slightly decreased E/I ratio after fear conditioning (from ≈ 0.8 to 0.6), whereas Firoc mice show a 3-fold increase in E/I ratio. It seems that a diminished E/I ratio before fear learning leads to an exaggerated increase in E/I ratio in parallel with exaggerated fear expression (freezing). In other words, a preserved IGF-I input before fear conditioning impedes an exaggerated increase in the E/I ratio afterwards. Furthermore, IGF-I blocks exaggerated fear expression in PTSD wild type mice when administered after fear learning. Although more experiments are needed (see below), we postulate that IGF-I enables coping behaviors by balancing the engagement of orexin neurons in responses to stress. This mechanism probably forms part of the homeostatic regulation of these neurons [47].

The observed adaptations of GABA and Glu synaptic structure onto orexin neurons suggest changes in their activity by increasing/decreasing the number of excitatory and inhibitory synaptic inputs. Additional modifications of intrinsic excitability of orexin neurons and/or of the efficacy of synaptic inputs through functional plasticity could also be contributing. Excitatory/inhibitory (E/I) input re-arrangements onto orexin neurons have been seen during the sleep phase in mice [45], suggesting a functional impact of the observed E/I plasticity, which agrees with increased δ band activity in the electroencephalogram of naive Firoc mice [16]. Conversely, under basal conditions, IGF-I slightly favors glutamatergic transmission onto orexin neurons, as indicated by moderately increased active post-synaptic glutamatergic receptors (increased double-labeled orexin/pSer^845^GluR1 neurons), whereas GABAergic transmission is downregulated, as indicated by reduced active post-synaptic GABA receptors (decreased pSer^783/893^ GABA(B) receptor). The intermediary role of AMPK in modulatory actions of IGF-I on orexin neurons (as indicated by decreased pThr^172^ AMPKα staining in these neurons), was partially confirmed through systemic modulation of AMPK activity using an agonist (AICAR) or an antagonist (CC) of this kinase. CC administration mimicked the effect of IGF-I in vivo and rescued the Firoc phenotype. Conversely, AICAR abrogated the rescuing actions of IGF-I in PTSD wild type mice. Experiments with timed in vivo down-regulation of AMPK activity in orexin neurons of PTSD-mice would help confirm that AMPK activity specifically in these neurons is sufficient to inhibit PTSD expression. Of note, the related hormone insulin inhibits also hypothalamic AMPK activity [48].

A reduced E/I ratio in hippocampal neurons of serum IGF-I deficient mice was previously documented by us [49], suggesting a broader modulation by IGF-I of E/I balance. Indeed, this same modulatory effect on E/I transmission has recently been postulated for invertebrate insulin peptides [50], and confirmed by us in the mouse cerebral cortex [51]. A recent report in obese mice shows a similar E/I imbalance in orexin neurons leading to a mood phenotype reminiscent of what we see in Firoc mice [52], reinforcing the notion that an imbalanced E/I ratio in orexin neurons hinders appropriate responses to stress. Altogether, these results expand previous observations suggesting that IGF-I is a neuroendocrine regulator of neuronal plasticity in different brain areas (reviewed in [53]), affecting in this case coping behavior.

IGF-I in orexin neurons modulates both acquired (fear learning), and innate fear (predator exposure) responses, providing a specific mechanism for resilience/ vulnerability to emotional trauma. Indeed, Firoc mice developed PTSD-like behaviors [54] after fear conditioning, display changes in gene expression in various brain areas reminiscent of changes seen in other experimental models of PTSD (not shown), and showed a decreased VGlut2/Vgat ratio onto the soma of orexin neurons, suggesting reduced glutamatergic (excitatory) inputs and increased GABAergic (inhibitory) inputs. Larger inhibitory effects of LPO stimulation in Firoc mice agree with the latter. In addition, PTSD-like behavior of Firoc or wild-type mice submitted to 2.5 mA electric shocks is normalized by early chemogenetic inhibition of orexin neurons, which supports the use of orexin receptor antagonists for treatment of this condition [20, 21]. Conversely, recent observations pose orexin activation in experimental PTSD as a mechanism of resilience [22], supporting the use of orexin agonists for treatment of PTSD [55]. These two apparently opposing observations may be reconciled by our findings. Since tempered responses to stress require appropriate IGF-I signaling onto orexin neurons, IGF-I may modulate orexin activity in response to fear stimuli to enable coping responses, as suggested by Cohen et al [22]. Indeed, after a strong fear stimulus, PTSD-like behavior in wild-type mice was corrected by early administration of IGF-I, a procedure that was not effective in Firoc mice lacking functional IGF-I receptors in orexin neurons. In turn, when orexin regulation by IGF-I is compromised (as in Firoc mice), orexin activity becomes maladaptive; i.e., greater expression of orexin, increased number of double-labeled c-fos^+^/orexin^+^ and c-fos^+^/TH^+^ cells, and markedly increased E/I ratio onto orexin neurons are found in Firoc mice after fear conditioning, as compared to littermates. Thus, exaggerated activation of the orexin-LC circuitry leading to aberrant fear behavior [32] would be produced by lack of IGF-I balancing. The reason why PTSD-like behavior is elicited in wild-type mice in response to a stronger stress (2.5 mA electric shocks), and ameliorated by systemic IGF-I administration may be related to the fact that stress hinders brain IGF-I input [56]. Again, reduced IGF-I regulation of orexin activity would underlie this process. In this vein, low levels of serum IGF-I may be associated with increased vulnerability to stress [5].

This novel interaction between IGF-I and orexin activity has allowed us to document that early intervention abrogates long-term PTSD traits such as impaired fear extinction and anhedonia. This furthers previous observations that early treatment provides better therapeutic efficacy in PTSD [57] and that an imbalanced E/I ratio is underlying this condition (https://www.ptsd.va.gov/professional/treat/txessentials/clinician_guide_meds.asp#biodisturb). Indeed, early inhibition of excess orexinergic activity in Firoc mice by DREADDi or CC, or in wild type mice submitted to intense fear learning with either DREADDi, systemic IGF-I administration, or CC, corrected PTSD-like traits. Since using these three different short-term treatments within a given time window leads to prolonged effects, we consider that a context-dependent re-wiring of involved circuits underlies the observed effects. Although AMPK and IGF-I exert multiple roles in different cell types, the possibility of using AMPK inhibitors/IGF-I analogs for treatment of PTSD may be worthy to explore with caution.

The fact that a pleiotropic neurotrophic factor such as IGF-I [53] modulates the activity of orexin neurons, considered also a multitasking system [28], contributes to explain the diversity of actions of IGF-I in the brain, including regulation of mood. Indeed, numerous reports relate IGF-I with affective disorders [7, 13, 58–61], and specific potential mechanisms have started to unfold. For example, recent observations indicate that IGF-I participates in depression through its role in the serotonin system [6], while others related it to its involvement in adult hippocampal neurogenesis [62] or blood-brain-barrier function [63, 64]. Thus, our observations widen the number of potential mechanisms whereby IGF-I intervenes in mood homeostasis, including coping responses through the orexinergic system, which emphasizes the system level (i.e.,: mood) of actions of this growth factor in the brain.

Our study has several limitations. For instance, we have not analyzed the possible role of sleep disturbances in the PTSD-like phenotype displayed by Firoc mice after fear-learning since naïve Firoc mice show increased sleepiness during the inactive period [16]. It might well be that they develop further sleep disturbances. We have not determined either the possible involvement of other brain areas, such as the amygdala, of high relevance in PTSD [65]. Indeed, IGF-I participates at cortical level in fear expression [9]. Also, whether IGF-I modulates the activity of other hypothalamic neurons involved in fear responses, such as oxytocin neurons in the paraventricular hypothalamic nucleus [14], remains to be explored. Further experiments looking at changes in E/I ratio using slice recordings in PTSD wild type mice and the effects of IGF-I treatment would give greater insight into what appears to be a context-dependent effect of IGF-I on orexin neurons.

In summary, inhibition of AMPK by IGF-I modulates E/I balance onto orexin neurons, a hypothalamic neuron that participates in behavioral responses to fear. Accordingly, inappropriate systemic IGF-I input onto orexin neurons may interfere with balanced coping behaviors, which provides novel targets for therapy of stress disorders such as AMPK.

## Materials and Methods

### Animals

Adult female and male C57BL/6 J mice (3–4 months, Harlan Laboratories, Spain), and Cre/Lox mice lacking IGF-I receptors in orexin neurons (Firoc mice) were used in all experiments in a sex-balanced manner without ramdomization. No sex-specific differences were found in these studies. Adult male Wistar rats were used in predator exposure tests. Firoc mice were obtained by crossing Orexin-Cre mice (a kind gift of Dr T Sakurai, Tsukuba Univ, Japan; [66]) with IGF-IR^f/f^ mice (B6, 129 background; Jackson Labs; stock number: 012251) as explained in detail elsewhere [16]. Orexin neurons in Firoc mice show attenuated responses to systemic IGF-I administration, as assessed by c-fos and phospho-Akt expression [16].

Genotyping of Firoc mice was performed using 5´-GGTTCGTTCACTCATGGAA AATAG-3´, and 5´-GGTATCTCTGACCAGAGTCATCCT-3´ for Orexin-Cre and 5´-CTTCCCAGCTTGCTACTCTAGG-3´, and 5´-CAGGCTTGCAATGAGACATGGG-3´ for IGF-IR^f/f^. DNA from brain tissue was isolated using Trizol Reagent and ethanol precipitation. 10 ng of genomic DNA was used in a PCR reaction containing 1X reaction buffer, 1 µM of each primer, 0.2 mM of dNTPS, and 0.75ul of DFS-Taq DNA polymerase (Bioron, GmbH). The thermocycler program was 92 °C, 3 min and 30 cycles of 94 °C, 30 sec; 65 °C, 30 sec; 72 °C, 30 s, after that a final extension step at 72 °C for 2 min was performed. Amplicons were analyzed in 3% agarose gels stained with SYBRsafe (Thermofisher, Inc).

Animals were housed in species-specific standard cages (mice 5 per cage; rats 1–2 rats per cage) and kept in a room with controlled temperature (22 °C) under a 12–12 h light-dark cycle. All animals were fed with a pellet rodent diet and water *ad libitum*. All experimental protocols were performed during the light cycle. Mice were handled for 3 days prior to any experimental manipulations. Animal procedures followed European guidelines (2010/63EU, European Council Directive) and were approved by the local Bioethics Committee (Government of the Community of Madrid, PROEX 193.4/20). Sample sizes were kept as little as possible to comply with current animal reduction policies.

### Viral constructs

For chemogenetic experiments using DREADD, a viral construct (AAV-hSyn-DIO-hM4D(Gi)-mCherry; AAV5; 8.6 × 10^12^ viral infective units/ml) was locally injected bilaterally to inactivate orexin-cre neurons in Orexin-Cre (littermates) and transgenic mice (Firoc). As control virus, we used AAV-hSyn-DIO-mCherry (AAV5; 5 × 10^12^ viral infective units/ml). Both viral constructions were obtained from Addgene (pAAV-hSyn-DIO-hM4D(Gi)-mCherry (AAV5), # 44362-AAV5 and pAAV-hSyn-DIO-mCherry (AAV5) # 50459-AAV5). Clozapine N-Oxide (CNO, 2 mg/kg dissolved in saline 0.9%) was administered ip and 40 min later behavior was assessed, CNO efficacy in orexin neurons was confirmed in acute slices obtained from injected mice. Slices for electrophysiological recordings were prepared from 2-months old Orexin-Cre mice, 4 weeks after injection of the DREADD-mCherry virus into the lateral hypothalamus. Brains were quickly removed and coronal slices (250 µm) containing the lateral hypothalamus were cut with a vibratome (4 °C) in a solution containing: 234 mM sucrose, 11 mM glucose, 26 mM NaHCO_3_, 2.5 mM KCl, 1.25 mM NaH_2_PO_4_, 10 mM MgSO_4_, and mM 0.5 CaCl_2_ (equilibrated with 95% O_2_–5% CO_2_). Recordings were obtained at 30–32 ^o^C from orexin^+^ neurons identified using fluorescence microscopy (mCherry^+^) in oxygenated artificial cerebrospinal fluid containing the following: 126 mM NaCl, 26 mM NaHCO_3_, 2.5 mM KCl, 1.25 mM NaH_2_PO_4_, 2 mM MgSO_4_, 2 mM CaCl_2_ and 10 mM glucose (pH 7.4). Patch-clamp electrodes contained intracellular solution composed of 131 mM K gluconate, 5 mM KCl, 4 mM MgCl2, 10 mM HEPES, 4 mM EGTA, 2 mM MgATP, and 0.3 mM Na_2_GTP (pH 7.3) corrected with KOH (290 mOsm). Positive and negative currents were injected during 600 ms to calculate action potential frequency and input resistance. CNO (2 µM) was applied through perfusate dissolved in ACSF. Signals were amplified, using a Multiclamp 200B patch-clamp amplifier (Axon Instruments, Foster City, California, United States), sampled at 20 kHz, filtered at 10 kHz, and stored on a PC. Data were analyzed using pClamp (Axon Instruments). CNO reduced input resistance and firing frequency of orexin neurons from Orexin-Cre mice transduced with pAAV-hSyn-DIO-hM4D(Gi)-mCherry: Input resistance relative change; baseline 1.04 + 0.02; CNO = 0.76 + 0.13 (two tailed Mann Whitney test, *U* = 0, *p* = 0.03); firing frequency relative change; baseline 0.96 + 0.02; CNO = 0.33 + 0.2 (Two tailed Mann Whitney test, *U* = 0, *p* = 0.02, *n* = 4 neurons from 4 acute slices obtained from 2 mice). Only one neuron was recorded in each slice. CNO effect was quantified 5 m after application started (Suppl Fig. 3).

For inactivation of IGF-IR in orexin neurons of adult mice, we bilaterally injected in the lateral hypothalamus (stereotaxic coordinates: AP = −1.4; ML = ± 0.9; DV = −5.4) of IGF-IR^f/f^ mice (3 months-old), a AAV-Orexin-Cre-EGFP (5 ×10^12^ viral infective units/ml) virus (AAV-Firoc) or a AAV-CMV-EGFP (1.3 ×10^13^ viral infective units/ml) virus (AAV-CMV-Control), and 1 month later we submitted them to fear-learning (see below). Also, a group of littermates was injected with AAV-Orexin-Cre-EGFP (AAV-Control). Since both control groups gave similar behavioral responses they were pooled together as a single AAV-Control group. In addition, the percentage of infected orexin cells was similar in both control groups (Suppl Fig. 2A). The HCRT mini-promoter (Ple112; [67] used in this construction) was obtained from plasmid pEMS1418 (Addgene #29214) by enzymatic restriction digestion and inserted into a pAAV-CMV-CRE-ires-GFP-WPRE plasmid, in a cloning step that implies the substitution of the CMV promoter by the HCRT mini-promoter. The resulting plasmid pAAV-HCRT-CRE-ires-GFP-WPRE was used for AAV particles packaging (Viral Vector Production Unit, UAB-VHIR, Barcelona, Spain.).

### Surgery

For all surgeries, mice were anaesthetized with isoflurane (Zoetis) administered with a nose mask (David Kopf Instruments, France), and placed on a stereotaxic frame (Stoelting Co) on a heating pad and tape in their eyes to protect them from light. For viral expression, mice were injected with a 5 µl Hamilton syringe bilaterally into the orexin nuclei (AP = −1.4; ML = ± 0.9; DV = −5.4) 4 weeks before experiments. For DREADD viral expression, 300 nl were bilaterally infused at a rate of 100 nl/min and the Hamilton syringe withdrawn 10 min later. For slice electrophysiological recordings 4-week-old mice were used for DREADD surgeries.

### Behavioral tests

#### Cued-fear conditioning

We used classical fear conditioning as it is considered a robust model of PTSD [23]. Experiments were performed at the same time of the day during the light phase with 3–4 months-old male and female mice (*n* = 14–19 mice/genotype, balanced sex). The training and test sessions were video recorded. Mice were placed in a shuttle box chamber (AccuScan Instruments) for 120 s before exposing them to a neutral conditioned stimulus (CS+) -a tone of 80 dB for 30 s, presented together with an aversive unconditioned stimulus (US) -an electrical footshock of 0.3 mA/2 s, except in the case of the PTSD-eliciting protocol, where 2.5 mA shocks were used [34]. Training consisted of 5 consecutive trials with 90 s intertrials (CS−). Animals remained in the chamber for 5 min more before returning them to their home cages. Twenty-four hours later, fear conditioning was tested placing the animals in a different context using the same protocol but without the unconditioned stimulus (US). As a consequence of the US–CS association, mice displayed freezing behavior, which was scored for every trial (CS+), and intertrial (CS−). Freezing behavior was defined by the absence of movement except of breathing and heart beating. Context-dependent extinction was tested 1 and 5 weeks later (delayed extinction). Mice were placed in the same context for 5 min to measure their freezing behavior. The latter unveils PTSD-like behavior when it is abnormally retained.

#### Predator exposure test

Control littermates and Firoc mice implanted with osmotic minipumps (either with saline or IGF-I, see below), were introduced 3 days later into a box for 10 min with two compartments separated by a plastic grid, one containing a rat (predator) and the other empty. The box contained bedding of the rat with urine and feces. All sessions were recorded, and every rat was exposed to a maximum of 4 mice/day. Grid contacts, freezing time and bedding burying behaviors of exposed mice were scored as a measure of anxiety [5].

#### Elevated plus maze

We assessed anxiety-like behavior in the elevated plus maze, following previously published procedures [5]. The test measures anxiety as a function of time spent in the open arms vs the closed arms of the maze. Time and entries in open and closed arms were scored; more time spent in the open arms indicate less anxiety levels. Two days after exposure to the rat, mice with implanted minipumps were introduced in a maze of 40 cm from the floor with two opposing protected (closed) arms of 30 cm (length) × 5 cm (wide) × 15.25 (height), and two opposing unprotected (open) arms of 30 cm (length) × 5 cm (wide). Each animal was introduced in the center of the maze for 10 min. All measures were recorded with an automated video-tracking system (Video Tracking Plus Maze Mouse; Med Associates, USA).

#### Sucrose preference test

This test is used to measure anhedonia, a lack of proper response to positive stimuli [68]. Mice were given 2 bottles of water for 3 days, and then 2 bottles of 2% sucrose for 2 days. Afterwards, mice were deprived of food and water for 18 h and then, they were presented a bottle of water and a bottle of 2% sucrose during 2 h. The position of the bottles was switched after 1 h. Bottles were placed in the active phase. Water and sucrose consumption was recorded, and sucrose preference was defined as the ratio of the volume of sucrose intake to the volume of total intake of liquid (water and sucrose).

#### Escape test

This test measures defensive behavior [69]. Mice were introduced in a shuttle box chamber (AccuScan Instruments) which is separated in two compartments. The test comprises 5 trials, separated by inter-trials of 30 s. Each trial consisted of an escapable foot shock of 10 s/0.1 mA which stops when the animal crosses to the opposite compartment [70]. This behavior is learned easily when more trials are performed [71]. Latencies to escape from the shock were quantified with Versamax software.

### Immunocytochemistry

Antibodies used are shown in Supplementary Table 1. Mice were perfused trans-cardially under deep anesthesia (sodium pentobarbital, 50 mg/kg, intraperitoneously) with 100 ml of saline buffer 0.9% followed by 100 ml of 4% paraformaldehyde (PFA) in 0.1 N, pH 7.4 phosphate buffer (PB), 90 min after the cued-fear conditioning test, the recall context, or under basal conditions. This time point was selected as optimal to see c-Fos staining. For experiments with systemic administration of IGF-I, animals were sacrificed 2 h after injection. Brains were removed, post-fixed overnight at 4 °C in the same fixative, and cut at 50 µm thick sections on a vibratome (Leica VT 1000 S). Sections were kept at 4 °C, immersed in 0.1 N PB with 0.02% sodium azide, until processing. Serial coronal free-floating sections were rinsed in 0.1 N PB for 10 min, and a blocking solution containing 10% normal donkey serum, and 0.4% Triton X-100 in 0.1 N PB (PB-T) was added and maintained at room temperature for 2 h. Thereafter, sections were incubated overnight at 4 °C in the same solution with the corresponding primary antibodies (see Antibody Table). The next day, sections were washed 3 times with PB-T, and incubated with secondary antibodies (see Suppl Table 1) for 2 h at room temperature. After the incubation, slices were washed 3 times with PB-T and incubated 5 min with Hoechst (1:500 dilution, Life Technologies). Finally, sections were washed 3 times with PB and mounted onto glass slides coated with gelatin in Gelvatol mounting medium. For double immunostaining of MCH/ pGABA(B)R2, when antibodies were done in the same host, we performed a sequential immunolabeling. Firstly, as previously described, we incubated with anti-MCH antibody and his corresponding secondary antibody. Sections were washed 3 times with PB-T, fixed with 1% PFA for 10 min and blocked again for 1 h. Then, tissue was incubated with primary anti-pGABA(B)R2 antibody overnight and thereafter with its corresponding secondary antibody. Final steps were as above. Images were taken with confocal microscopy (SP5, Leica Microsystems, Germany).

### Image analysis

Analyses were carried out with Imaris software (Bitplane), which allows to examine images in 3D. With the images taken at 20x magnification, we created spots with an estimated XY size of 15 µm to count orexin^+^ or TH^+^ neurons in their channel (red or pseudo-color in red for orexin labeling in DREADD experiments), and next we added a filter corresponding to the c-Fos channel (green) to calculate the number of orexin^+^/c-Fos^+^ and TH^+^/c-Fos^+^ cells. To score excitatory and inhibitory synaptic inputs in orexin neurons we performed triple immunocytochemistry with VGLUT2 (excitatory) and VGAT (inhibitory) antibodies (see Suppl Table 1). As explained elsewhere by others [72], we created a surface in the images taken at 63x magnification corresponding to all orexin neuron surfaces in their channel (pseudo-color gray). Then, to filter the VGAT (green) and VGLUT2 (pseudo-color red) channels, we masked them in the orexin surface, to only maintain that portion of the channels. Orthogonal views of images were taken to assure that identified spots were on orexin^+^ cells (Suppl Fig. 4F). Last, we created spots for VGAT and VGLUT2 with an estimated XY size of 0.5 µm, and we counted the number of VGAT and VGLUT2 spots in orexin^+^ cells. Similar procedures were used when evaluating double-labeled (orexin or MCH) neurons with pSer^783/893^GABAR(B), pSer^845^GluR1, and pThr^172^AMPK.

### RNAscope

RNAscope (2.5 HD Detection kit—Red; #322350; ACD, USA) with an IGF-IR exon 3-specific probe combined with immunocytochemistry with anti-orexin antibody was also performed to confirm the deletion of exon 3 in orexin cells after virus injection

### qPCR

Animals were anesthetized with pentobarbital (50 mg/kg, ip). After dissecting the brain, the different brain areas (hypothalamus, hippocampus, prefrontal cortex) were collected and frozen at -80 °C until use. Tissue RNA was extracted with Trizol (Life Technologies, USA), as described elsewhere [73]. cDNA was synthesized from 1 μg of RNA of each sample following the manufacturer’s instructions (Hight Capacity cDNA Reverse Transcription Kit; Applied Biosystems). Fast Real-time qPCR was performed using the SYBR Green method (Fast SYBR Green Master Mix, Applied Biosystems) with the QuantStudio 3 Real-Time PCR System (Applied Biosystems). Relative mRNA expression was determined by the 2^−ΔΔCT^ method [74], and normalized to GAPDH levels. All primers were commercial (or predesigned) from Themo Fisher (see Suppl Table 2).

### Drug administration

Human recombinant IGF-I (Pre-Protech, USA) or vehicle (saline) were administered intracerebroventricularly (icv, 1 µg/day) using Alzet osmotic mini-pumps for 7 days (Model 1007D), and the brain infusion kit 3 (Alzet, USA) with the following stereotaxic coordinates: AP = −0.5; ML = 1.1; DV = −2.5, as indicated before [5]. Pumps were implanted subcutaneously between the scapulae. In other experiments IGF-I was given intraperitoneally (ip, 1 µg/gr) [16] to wild type mice submitted to the 2.5 mA fear-learning protocol 6 h after fear conditioning. For hdM4Di (DREADD) experiments, clozapine-N-oxide (CNO, Tocris) was administered at 2 mg/kg dissolved in saline 0.9%, and injected intraperitoneally 40 min before test sessions, and 24 h and 1week after fear conditioning. 6-[4-(2-Piperidin-1-yl-ethoxy)-phenyl]-3-pyridin-4-yl-pyrrazolo [1,5-a]-pyrimidine dihydrochloride (Compound C dihydrochloride, AMPK inhibitor; Cat. # CD0339, Chemdea NJ, USA) was administered intraperitoneally (ip, 10 mg/kg) [75] 6 h after fear conditioning to PTSD-like wild type mice. 5-Aminoimidazole-4-carboxamide ribonucleotide (AICAR, AMPK activator, Cat. # A611700, Toronto Research Chemicals ON, Canada) was administered intraperitoneally (ip, 500 mg/kg) [75] to wild type mice submitted to the 2.5 mA fear-learning protocol 30 min after a previous IGF-I ip injection (given 6 h after the shocks). Different timings used for drug administration were based on prior experience.

### Recordings in anesthetized animals

Electrophysiological recordings were performed as described [16]. Briefly, Control and Firoc mice were anesthetized with isoflurane (2% induction; 1–1.5% in oxygen, maintenance doses), placed in a David Kopf stereotaxic apparatus (Tujunga, CA, USA) in which surgical procedures and recordings were performed, with a warming pad (Gaymar T/Pump, USA) set at 37 °C. Local anesthetic (lidocaine 1%) was applied to all skin incisions and pressure points. An incision was made exposing the skull, and small holes were drilled in the skull. An optrode (Optical fiber + Tungsten microelectrode of 0.5–0.8 MΩ, with a core diameter 120 µm; Thomas Recording) was used to record the evoked potential in the LH/PeF area (coordinates from Bregma: A,−1.95; L, 1.0 and depth, 4.0–4.5 mm). Unitary recordings were performed through the Tungsten microelectrode of the optrode. Signal was filtered (0.3–3 kHz) and amplified using a DAM80 preamplifier (World Precision Instruments). Electric stimulation was performed in the lateral preoptic area (LPO, single square pulses, 0.3 ms duration, and 20-50 µA intensity, delivered at 1 Hz; Cibertec Stimulator, Spain) with a delay of 100 ms before blue-light stimulation to induce inhibition in the LH/PeF area. The light-evoked unit activity in the LH/PeF in basal condition was compared to the light-evoked activity after LPO stimulation.

### Optogenetics

Optogenetic experiments were performed to identify orexin neurons through light activation using mice expressing channelrhodopsin under the orexin promoter (Ox-ChR mice) as controls and Firoc-ChR mice, as described [16]. Animals were anesthetized with isoflurane, positioned in the stereotaxic apparatus, and handled as above. The scalp’s sagittal midline was sectioned and retracted, and a small craniotomy was drilled over the Perifornical (LH/PeF) hypothalamic area (same stereotaxic coordinates as above). Optical stimulation of ChR-expressing neurons was achieved with light-emitting diodes (LED; 300 ms pulse; Thomas Recording, Germany). Multi- and single-unit recordings were performed through the optrode, filtered (0.3–3 kHz), and amplified using a DAM80 preamplifier (World Precision Instruments). Multi-unit and single-unit activities were sampled with the aid of Spike2 software (Cambridge Electronic Design, UK) at 10 kHz via an analog-to-digital converter built into the Power 1401 data acquisition unit and fed into a PC for off-line analysis with Spike 2 software. Unit activity was extracted from the recording using a filter from 0.3–3 kHz. Multi-unit and single-unit responses elicited by LC stimulation in the LH/PeF area were calculated using a per-stimulus time histogram (1 ms bin). A square-step voltage command triggered the LED. A single long-lasting pulse was applied (473 nm light, 26 stimuli with a duration of 300 ms) with an illumination intensity of < 30 mW/mm2, below the damage threshold of ~100 mW/mm2 for blue light [76]. LPO electrical stimulation with single square pulses to elicit inhibition in LH/PeF area was carried out 100 ms before light stimulation (0.3 ms duration, and 20–50 µA intensity, delivered at 1 Hz; Cibertec Stimulator, Spain). In an off-line analysis, the single-unit activity during the light pulse in the first 50 ms was subtracted from the basal activity (50 ms before the light). Neurons that have a response of fewer than 0.2 spikes per stimulus were ruled out. Several stimuli were selected to avoid spontaneous oscillations’ artifact. The stimuli have been considered a clean response, as long as there are more than six stimuli continuously. We compared basal unitary activity (light pulse stimulation) with the second stimulation (LPO electrical stimulation + light pulse stimulation, with 100 ms delay between them).

### Statistics

Statistical analysis was performed using Graph Pad Prism 8 software (San Diego, CA, USA). The sample size for each experiment was chosen based on previous experience and considering a reduced use of animals. No blinding of data was used. All data were included for analysis. When data followed a normal distribution, we used student’s t-test for comparing two groups (Welch’s correction was used for data sets with unequal variance), and for more than two groups either 1-way, 2-way or 3-way ANOVAs or 2-way RM ANOVAs followed by Tukey’s or Sidak’s multiple comparison test as a post hoc. For non-normally distributed data, we used the Mann–Whitney U. All results are shown as mean ± standard error (SEM) and significant values as: *p < 0.05; **p < 0.01; ***p < 0.001.

## Supplementary information

Supl Table 1

Supl Table 2

Legends Suppl Figs

Supl Fig 1

Supl Fig 2

Supl Fig 3

Supl Fig 4
