# Supplementary material for: Insulin-like growth factor I mitigates post-traumatic stress by inhibiting AMP-kinase in orexin neurons
Source: Mol Psychiatry. 2022 Feb 3;27(4):2182–96. doi: 10.1038/s41380-022-01442-9 (PMC9126821; doi:10.1038/s41380-022-01442-9)
Supplement: Supplementary file 1 — Supl Table 1 [file 41380_2022_1442_MOESM1_ESM.docx]

Antibodies used

| Primary antibody | Host | Dilution | Company |
| --- | --- | --- | --- |
| Anti-orexin-A | Mouse | 1:200 | Santa Cruz, sc-80263 |
| Anti-orexin-A | Rabbit | 1:1000 | Abcam, ab6214 |
| Anti-c-Fos | Rabbit | 1:2000 | Abcam, ab190289 |
| Anti-tyrosine hydroxilase | Mouse | 1:1000 | Millipore, MAB318 |
| Anti-VGLUT2 | Guinea Pig | 1:1000 | Synaptic Systems, 135 404 |
| Anti-VGAT | Mouse | 1:1000 | Synaptic Systems, 131 011 |
| Anti-GABBR2 (phospho S783) | Rabbit | 1:250 | Abcam, ab72447 |
| Anti-GluR1 (phospho Ser845) | Rabbit | 1:500 | Novus Biologicals, NB300-171 |
| Anti- Phospho-AMPKα (Thr172) | Rabbit | 1:200 | Cell Signaling, 2535 |
| Anti- melanocyte concentrating hormone (MCH) | Rabbit | 1:2000 | Abcam, ab274415 |

| Secondary antibody | Host | Dilution | Company |
| --- | --- | --- | --- |
| AlexaFluor 488 | Mouse | 1:1000 | ThermoFischer, A-21202 |
| AlexaFluor 488 | Rabbit | 1:1000 | ThermoFischer, A-21206 |
| AlexaFluor-594 | Mouse | 1:1000 | ThermoFischer, A-21203 |
| AlexaFluor 594 | Rabbit | 1:1000 | ThermoFischer, A-21207 |
| AlexaFluor 647 | Guinea Pig | 1:1000 | ThermoFischer, A-21450 |
| AlexaFluor 647 | Mouse | 1:1000 | ThermoFischer, A-31571 |
