# Supplementary material for: Insulin-like growth factor I mitigates post-traumatic stress by inhibiting AMP-kinase in orexin neurons
Source: Mol Psychiatry. 2022 Feb 3;27(4):2182–96. doi: 10.1038/s41380-022-01442-9 (PMC9126821; doi:10.1038/s41380-022-01442-9)
Supplement: Supplementary file 2 — Supl Table 2 [file 41380_2022_1442_MOESM2_ESM.docx]

**Supplementary Primer Table**. Primers used in qPCR analyses.

| **Gene** | **Forward primer** | **Reverse primer** |
| --- | --- | --- |
| OXR | CTTTCCTTCTACAAAGGTTCC | CTTTCCCAGAGTCAGGATAC |
| Nrg1 | TAGTCACAGCTGGAGTAATG | CTGAGGAAGCTGTTACATTC |
| GAPDH | GGTGAAGGTCGGTGTGAACG | CTGCCTCCTGGAAGATGGTG |
