# Supplementary material for: Insulin-like growth factor I mitigates post-traumatic stress by inhibiting AMP-kinase in orexin neurons
Source: Mol Psychiatry. 2022 Feb 3;27(4):2182–96. doi: 10.1038/s41380-022-01442-9 (PMC9126821; doi:10.1038/s41380-022-01442-9)
Supplement: Supplementary file 3 — Legends Suppl Figs [file 41380_2022_1442_MOESM3_ESM.docx]

**Supplementary Figure 1.** **A,** Anxiety levels in Firoc mice are similar to those in control littermates under basal conditions (n=24-30 mice/group; Mann Whitney U Test). **B,** Escape behavior in basal conditions was normal in Firoc mice (n=5-8 mice/group; t-test).**C,D,** Fear-conditioning test: freezing time was scored every inter-trial of 90s (CS-), and trial (CS+) of 30s, both during the acquisition (C), and cued phases (D) (n=14-19 mice/group; *p<0.05 and **p<0.01vs controls; Two-way-ANOVA followed by Tukey’s test). Firoc mice show increased freezing times during the cued phases.

**Supplementary Figure 2.** **A**, Double labelled orexin (red with Alexa Fluor 594)/GFP (green) cells, marked with arrows, in LH of AAV-orexin-Cre.EGFP injected IGF-IR^f/f^ mice (AAV-Firoc mice), AAV-orexin-Cre.EGFP injected in littermates (AAV-Orexin-Cre-Control) and AAV CMV EGFP injected littermates (AAV-CMV Control). Histogram: Double labelled orexin/GFP cells estimated as percentage of total orexin labeled cells in AAV-Control mice, CMV-Control mice and AAV-Firoc mice (n=3)*.* Scale Bar: 50 µm. **B,** Non-truncated IGF-IR (with the presence of his exon 3 using a specific RNAscope probe in red) was decreased in AAV- Firoc mice in GFP+ (green) /orexin+ (pseudocolor in gray with Alexa Fluor 647) cells (Z = 7.5072; ***p<0.001). Scale Bar: 25 µm. **C,** Double labelled orexin (green with Alexa Fluor 488)/mCherry (red) cells in LH of AAV- DREADDi mCherry injected Orexin-Cre mice. Histogram: Double labelled orexin/mCherry cells estimated as percentage of total orexin cells (n=3). Scale Bar: 50 µm

**Supplementary Figure 3. A-D,** CNO activity in orexin neurons. **A,** Membrane potential response to a -25 pA current step of an DREADDi-expressing LH neuron in Orexin-Cre mice previously injected with AAV-AAV5-DIO-DREADDi-mCherry. Traces represent the average of 3 consecutive sweeps before (Control, black) and after 2 µM Clozapine N-Oxide application (CNO, red). **B,** Relative change in input resistance (Rin) in response to CNO application (red arrow). CNO reduced input resistance: Input resistance relative change; baseline 1.04 + 0.02; CNO = 0.76 + 0.13 (two tailed Mann Whitney test, U = 0, p = 0,03). **C,** Firing in response to a depolarizing current step (+15 pA) before (Control, black) and after 2µM CNO application (CNO, red). Traces represent the superposition of 3 consecutive sweeps in each condition. **D,** Relative change in firing frequency in response to CNO application (red arrow). Scale bars, 100 ms, 10 mV. CNO reduced firing frequency relative change: baseline 0.96 + 0.02; CNO = 0.33 + 0.2 (Two tailed Mann Whitney test, U = 0, p = 0,02, n = 4 neurons from 4 acute slices obtained from 2 mice. Only one neuron was recorded in each slice. CNO effect was quantified 5 minutes after application started.

**Supplementary Figure 4. A,** Anhedonia in naïve Firoc mice was normal (n=9 mice/group; t-test). **B,** Treatment of Firoc mice submitted to fear learning (0.3 mA) with ip IGF-I (1µg/gr; n=8) 18 hours prior to testing did not alter exaggerated freezing responses. However, Firoc mice treated with CC (n=5) showed normal freezing behavior, which was lower that non-treated Firoc mice (**p<0.01 and ***p<0.001; One-way-ANOVA followed by Tukey’s test; non-treated Firoc mice group is the same shown in Figure 2). Scale bars: 50 μm. **C,** Optogenetic identification of orexin neurons in Control-ChR and Firoc-ChR mice with short-lasting BLUE light elicited a comparable significant increase in both groups (n=19-19/group; **p<0.01); Control-ChR: 134.9 ± 24.58 spikes/100ms (26 stimuli), and Firoc-ChR mice: 145.4 ± 20.92 spikes/100ms, (26 stimuli) compared to basal conditions (111.1 ± 22.16 spikes/100ms, and 117.1 ± 16.47 spikes/100ms, respectively). At 200ms, the response was still higher in Control-ChR (130.7 ± 27.87 spikes/100ms, 26 stimuli; 19-19/group; *p<0.05), and Firoc-ChR mice (136.4 ± 20.89 spikes/100ms, 26 stimuli; 19-19/group; *p<0.05). No differences between groups were detected (p=0.8089; Ordinary Two-way ANOVA, Sidak's Multiple comparison tests). **D,** Levels of Ngr-1 mRNA in the hypothalamus of Firoc mice are significantly increased after fear-learning compared to controls (n=4 mice/group, *p<0.05 and ***p<0.001. Two-way ANOVA followed by Tukey´s test). **E,** Double immunostaining of pSer^783/893^ GABA(B) receptor (red with Alexa Fluor 594) and MCH (green with Alexa Fluor 488) showed no changes in double-immunostained cells (n= 100 MCH neurons/mouse, 3 mice per group; p=0.7974; t-test) or number of pSer^783/893^ GABA(B) receptor spot/cell after IGF-I treatment (n= 100 MCH neurons/mouse, 3 mice per group; p=0.3851; Mann Whitney U Test). Scale bar: 25μm, and 5μm in magnification. **F,** Representative orthogonal image (right, bottom; vertical, horizontal lines are planes of optical section) of a triple-labelled orexin neuron confocal Z-stack to document overlapping of boutons (green and red) with the neuronal soma (white). Bar is 5 µm.
