# Supplementary figures and images for: Insulin-like growth factor I mitigates post-traumatic stress by inhibiting AMP-kinase in orexin neurons
Source: Mol Psychiatry. 2022 Feb 3;27(4):2182–96. doi: 10.1038/s41380-022-01442-9 (PMC9126821; doi:10.1038/s41380-022-01442-9)

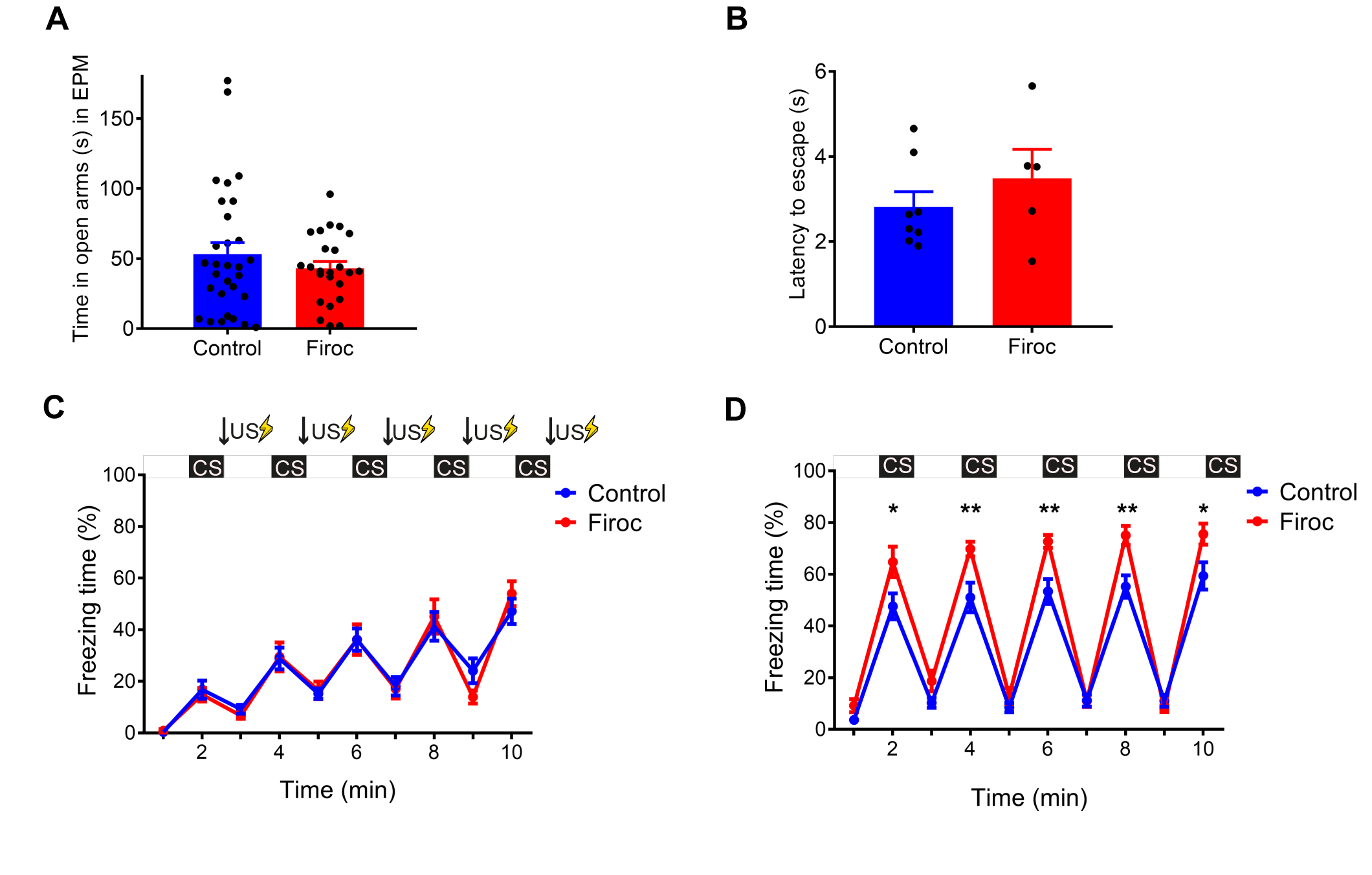

Supplement: Supplementary file 4 — Supl Fig 1 [file 41380_2022_1442_MOESM4_ESM.tif]

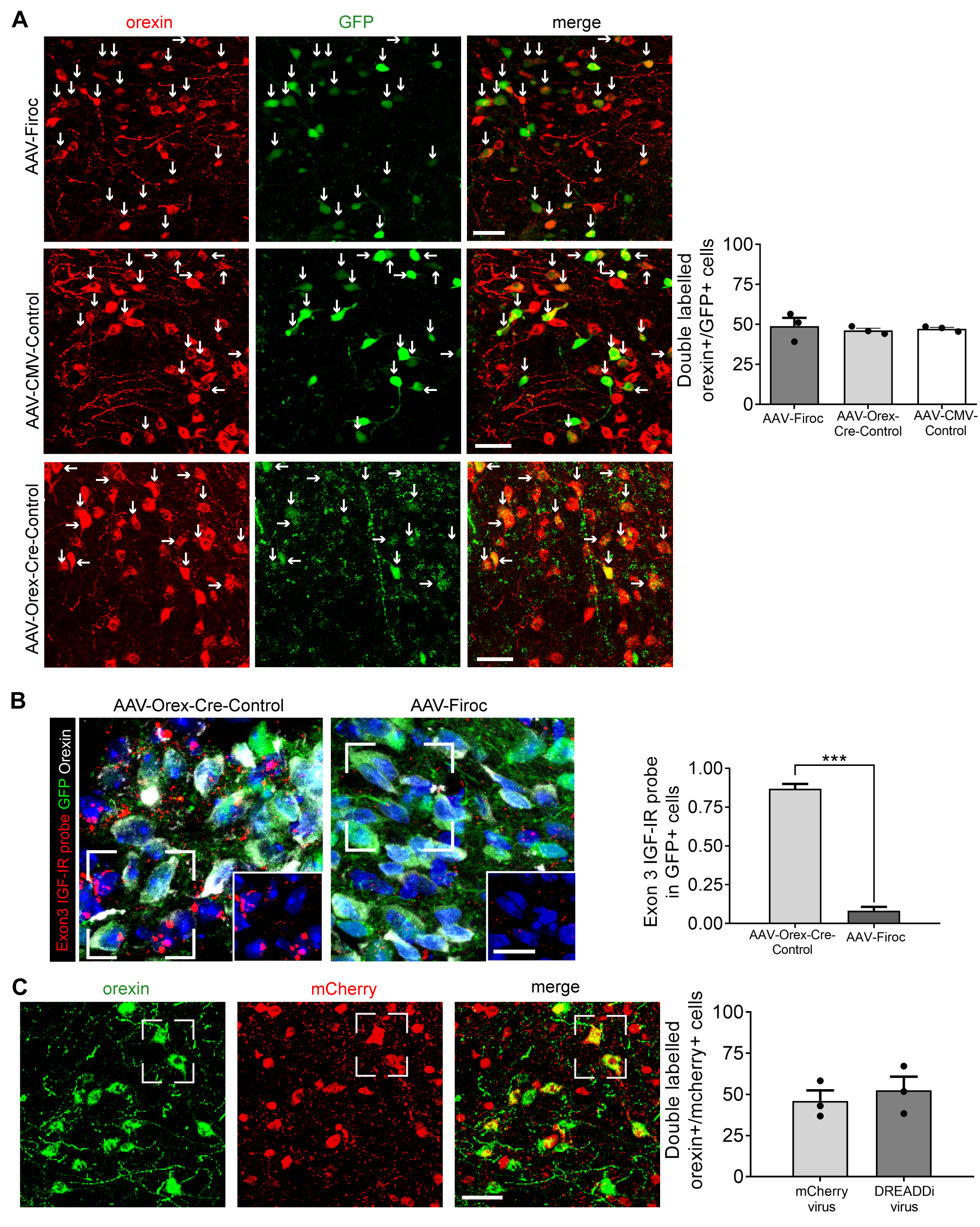

Supplement: Supplementary file 5 — Supl Fig 2 [file 41380_2022_1442_MOESM5_ESM.tif]

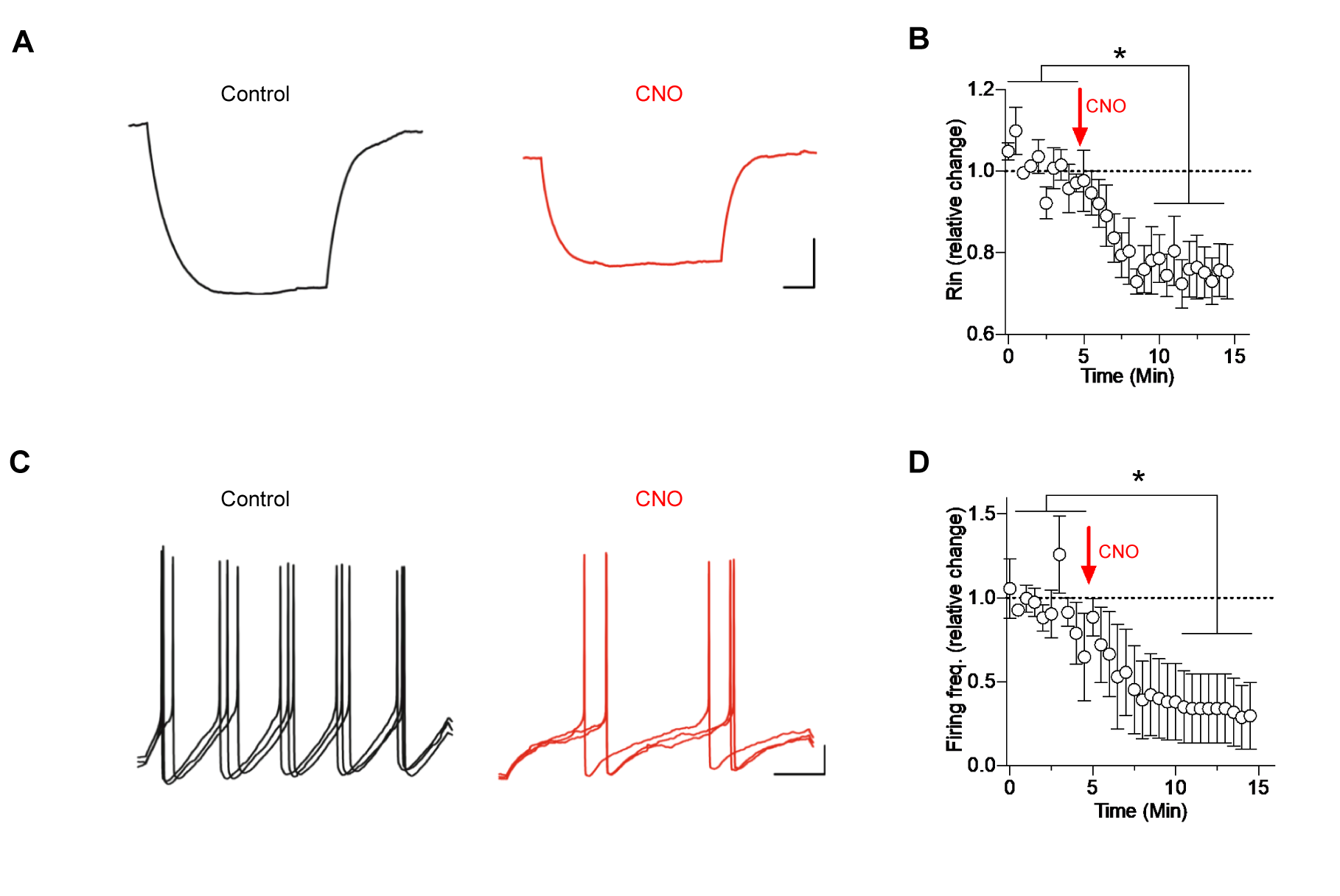

Supplement: Supplementary file 6 — Supl Fig 3 [file 41380_2022_1442_MOESM6_ESM.tif]

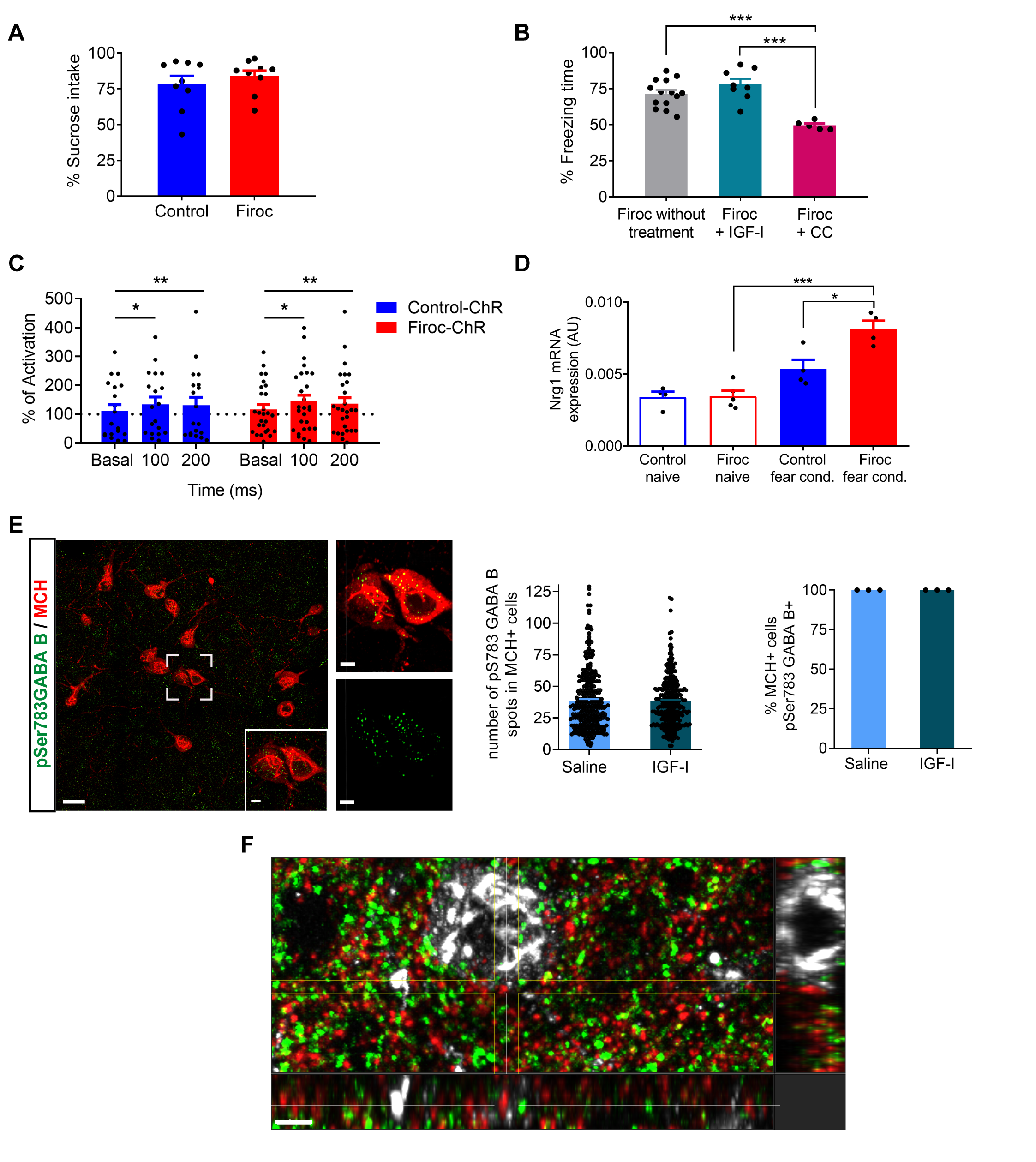

Supplement: Supplementary file 7 — Supl Fig 4 [file 41380_2022_1442_MOESM7_ESM.tif]
